# Dynamic genome wide expression profiling of *Drosophila* head development reveals a novel role of Hunchback in retinal glia cell development and blood-brain barrier integrity

**DOI:** 10.1371/journal.pgen.1007180

**Published:** 2018-01-23

**Authors:** Montserrat Torres-Oliva, Julia Schneider, Gordon Wiegleb, Felix Kaufholz, Nico Posnien

**Affiliations:** Universität Göttingen, Johann-Friedrich-Blumenbach-Institut für Zoologie und Anthropologie, Abteilung für Entwicklungsbiologie, GZMB Ernst-Caspari-Haus, Göttingen, Germany; New York University, UNITED STATES

## Abstract

*Drosophila melanogaster* head development represents a valuable process to study the developmental control of various organs, such as the antennae, the dorsal ocelli and the compound eyes from a common precursor, the eye-antennal imaginal disc. While the gene regulatory network underlying compound eye development has been extensively studied, the key transcription factors regulating the formation of other head structures from the same imaginal disc are largely unknown. We obtained the developmental transcriptome of the eye-antennal discs covering late patterning processes at the late 2^nd^ larval instar stage to the onset and progression of differentiation at the end of larval development. We revealed the expression profiles of all genes expressed during eye-antennal disc development and we determined temporally co-expressed genes by hierarchical clustering. Since co-expressed genes may be regulated by common transcriptional regulators, we combined our transcriptome dataset with publicly available ChIP-seq data to identify central transcription factors that co-regulate genes during head development. Besides the identification of already known and well-described transcription factors, we show that the transcription factor Hunchback (Hb) regulates a significant number of genes that are expressed during late differentiation stages. We confirm that *hb* is expressed in two polyploid subperineurial glia cells (carpet cells) and a thorough functional analysis shows that loss of Hb function results in a loss of carpet cells in the eye-antennal disc. Additionally, we provide for the first time functional data indicating that carpet cells are an integral part of the blood-brain barrier. Eventually, we combined our expression data with a *de novo* Hb motif search to reveal stage specific putative target genes of which we find a significant number indeed expressed in carpet cells.

## Author summary

The development of different cell types must be tightly coordinated, and the eye-antennal imaginal discs of *Drosophila melanogaster* represent an excellent model to study the molecular mechanisms underlying this coordination. These imaginal discs contain the anlagen of nearly all adult head structures, such as the antennae, the head cuticle, the ocelli and the compound eyes. While large scale screens have been performed to unravel the gene regulatory network underlying compound eye development, a comprehensive understanding of genome wide expression dynamics throughout head development is still missing to date. We studied the genome wide gene expression dynamics during eye-antennal disc development in *D*. *melanogaster* to identify new central regulators of the underlying gene regulatory network. Expression based gene clustering and transcription factor motif enrichment analyses revealed a central regulatory role of the transcription factor Hunchback (Hb). We confirmed that *hb* is expressed in two polyploid retinal subperineurial glia cells (carpet cells). Our functional analysis shows that Hb is necessary for carpet cell development and we show for the first time that the carpet cells are an integral part of the blood-brain barrier.

## Introduction

The development of complex organs is often accompanied by extensive cell- and tissue rearrangements. Initially simple cells undergo profound morphological changes such as extensive cell fusions of muscle precursor cells to form syncytial muscle fibers [1] or the formation of highly polarized neurons from initially uniform neuroectodermal cells [2,3]. Other cell types, such as germ cells, first migrate long distances before coming to rest in the developing gonads [4]. Although these cell-type specific processes need to be tightly controlled and coordinated with those of other cell types of the same and neighboring organs, the molecular mechanisms involved are still poorly understood. The development of the adult *Drosophila melanogaster* head and the visual system has been proven to be an excellent model to study the coordination of different developmental processes [5–10].

The adult *D*. *melanogaster* head is composed of the compound eyes (the main visual system), the three dorsal ocelli, the antennae, the ventral mouthparts and the head capsule that connects these organs and encloses the brain [11]. Most of these structures develop during larval stages from eye-antennal imaginal discs, which originate from about 20 cells that are specified at embryonic stages [12–14]. Throughout larval development, the eye-antennal discs grow extensively by cell proliferation resulting in discs composed of more than 15,000 cells at the beginning of pupation [7,15]. During the first two larval stages, the initially uniform disc is subdivided into an anterior antennal and a posterior retinal compartment by the action of two opposing morphogen gradients, which subsequently activate genes responsible for antennal development [16,17] and the retinal determination genes [15,18–24]. Approximately at the same time when the retinal part of the disc and the antennal region separate during the early L2 stage, the maxillary palp is defined in the ventral portion of the antennal part [25–27].

Once the eye-antennal disc is subdivided into the different organ precursors, cells within each compartment start to differentiate at L2/early L3 stages. In the retinal region, a differentiation wave that is established in the posterior most part of the equator region moves anteriorly. This wave is accompanied by a morphologically visible indentation, the so-called morphogenetic furrow (MF) [28]. In the region of the MF the expression of the proneural gene *atonal (ato)* becomes restricted to regularly spaced single cells posterior to the furrow [29,30]. Those cells are destined to become R8 photoreceptors, which subsequently recruit R1-R7 photoreceptors and associated cell types, such as cone and pigment cells from the surrounding cells [31–33].

The axons of successively forming photoreceptor cells need to be connected to the optic lobes to allow a functional wiring of the visual system with the brain. All axons are collected at the basal side of the eye-antennal disc and guided through the optic stalk throughout the L3 stage. This process is supported by retinal glia cells, which originate mainly by proliferation from 6–20 glia cells located in the optic stalk prior to photoreceptor differentiation [34–36]. These retinal glia cell types include migratory surface glia (including perineurial and subperineurial glia cells) and wrapping glia. Triggered by the presence of developing photoreceptor cells, the retinal glia cells enter the eye-antennal disc through the optic stalk and migrate towards the anterior part of the disc, always remaining posterior to the advancing morphogenetic furrow [34–36]. When photoreceptors differentiate, the contact of their growing axons with perineurial glia cells triggers the reprogramming of these glia cells into differentiated wrapping glia, which extend their cell membranes to ensheath bundles of axons that project to the brain lobes through the optic stalk [34,37,38]. The basally migrating perineurial glia cells and the wrapping glia ensheathed projecting axons are separated by two large polyploid carpet cells, each of them covering half of the retinal field [34]. The two carpet cells form septate junctions and express the G protein-coupled receptor (GPCR) encoded by the *moody* locus, both characteristics of the subperineurial surface glia type [34,39]. While subperineurial glia cells located in the brain remain there to form the blood-brain barrier, the carpet cells are thought to originate in the optic stalk [36], and during L2 and early L3 stages they migrate into the eye-antennal disc. Later during pupal stages, they migrate back through the optic stalk to remain beneath the lamina neuropil in the brain. However, so far it is not known, whether carpet cells or other retinal glia cell types eventually contribute to the formation of the blood-eye barrier, the retinal portion of the blood-brain barrier [40,41]. The carpet cells thus share features of subperineurial glia, but their extensive migratory behavior and their function in the eye-antennal disc suggest that these cells may exhibit distinct cellular features. However, so far, no carpet cell specific regulator has been identified that may be involved in specifying carpet cell fate.

Although eye-antennal disc growth and patterning, and especially retinal determination and differentiation, are among the most extensively studied processes in *D*. *melanogaster*, a systematic understanding of involved genes and their potential genetic and direct interactions is limited to the late L3 stage in the context of retinal differentiation [42–49]. Similarly, recent attempts to incorporate existing functional and genetic data into a gene regulatory network context covers mainly retinal determination and differentiation processes [50]. So far, a comprehensive profiling of gene expression dynamics throughout eye-antennal disc development is missing. The same holds true for the molecular control of retinal glia cell development. While the transcriptome of adult surface glia in the brain has been analyzed [51], retinal glia cells have not been comprehensively studied yet.

Here we present a dynamic genome wide expression analysis of *D*. *melanogaster* eye-antennal disc development covering late L2 to late L3 stages. We show that the transition from patterning to differentiation is accompanied by extensive remodeling of the transcriptional landscape. Furthermore, we identified central transcription factors that are likely to regulate a high number of co-expressed genes and thus key developmental processes in the different organ anlagen defined in the eye-antennal disc. One of these central factors is the C2H2 zinc-finger transcription factor Hunchback (Hb) [52] that has been extensively studied in *D*. *melanogaster* during early axis determination and segmentation [53,54]. It is also well-known for its role in the regulation of temporal neuroblast identity during embryogenesis, as it determines first-born identity in the neural lineage [55,56]. Here we show for the first time that *hb* is expressed in carpet cells and loss of function experiments suggest that its activity is necessary for carpet cell formation and/or migration and consequently for blood-brain barrier integrity. Eventually, we reveal putative Hb target genes and confirm that bioinformatically predicted targets are indeed expressed in developing carpet cells.

## Results

### Differential gene expression during *D*. *melanogaster* head development

**Fig 1:**
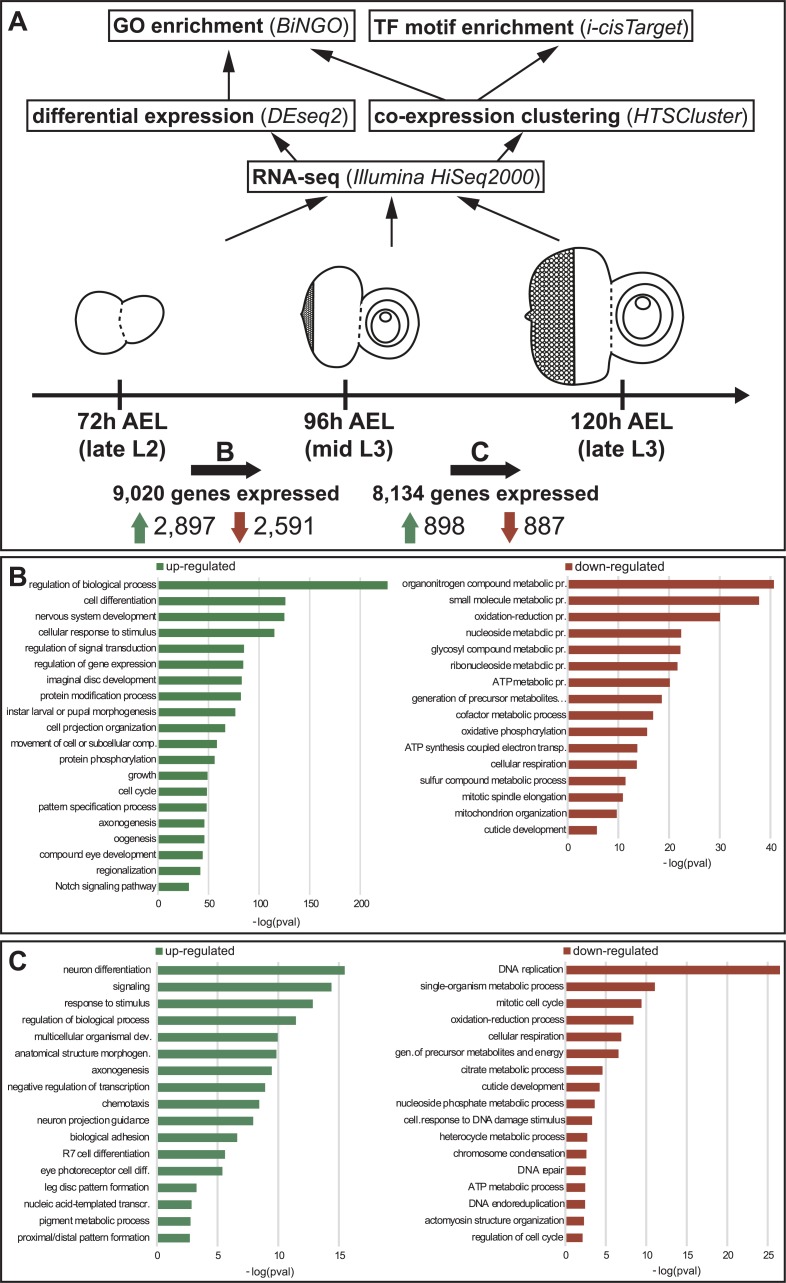
Analysis pipeline and expression dynamics at stage transitions. (A) The mRNA of eye-antennal imaginal discs was sequenced at three developmental stages: late L2, mid L3 and late L3. Upwards arrows illustrate the applied analysis pipeline. The total number of expressed genes for each stage transition is shown below the imaginal discs (**B:** late L2 to mid L3; **C:** mid L3 to late L3). The numbers next to the green arrows represent upregulated genes in each transition and the numbers next to the red arrow represent downregulated genes. (B) GO term enriched in the upregulated (green, left) and downregulated (red, right) genes in the transition from late L2 to mid L3 stage. (C) GO terms enriched in the upregulated (green, left) and downregulated (red, right) genes in the transition from mid L3 to late L3 stage.

Although compound eye development and retinal differentiation are among the most intensively studied processes in *D*. *melanogaster*, a comprehensive understanding of the underlying gene expression dynamics is still missing to date. To identify the genes expressed during *D*. *melanogaster* eye-antennal disc development and their expression dynamics, we performed RNA-seq on this tissue at three larval stages covering the process of retinal differentiation that is marked by the progression of the morphogenetic furrow. The late L2 stage (72h after egg laying, AEL) represents the initiation of differentiation, at mid L3 stage (96h AEL) the morphogenetic furrow is in the middle of the retinal field and the late L3 stage (120h AEL) represents the end of morphogenetic furrow progression (Fig 1A). Multidimensional scaling clustering clearly indicated that the largest difference in gene expression (dimension 1) was between L2 eye-antennal discs (72h AEL) and L3 eye-antennal discs (96h and 120h AEL) (S1 Fig).

After filtering out not expressed and very lowly expressed genes, we observed that 9,194 genes were expressed at least in one of the three sequenced stages. As anticipated by the multidimensional scaling plot (S1 Fig), the number of genes that changed their expression between 72h AEL and 96h AEL was much larger than between 96h AEL and 120h AEL, (Fig 1A). In only 24 hours, during the transition from L2 to L3, 60% of the expressed genes changed their expression significantly. In the transition from mid L3 to late L3, in contrast, only 22% of the genes underwent a change in their expression. The observation that many more genes were significantly differentially expressed during the transition from late L2 stages to mid L3 stages may in part also be caused by the fact that only female discs were analyzed between 96h and 120h AEL, while we compared mixed males and females at 72h AEL with only females at 96h AEL during the first transition. Since about one third of all genes in *D*. *melanogaster* show signs of sex-specific expression [57], the differentially expressed genes in the first transition may also include some male or female biased genes. However, in a GO term analysis, we only found two terms associated with the search term “sex” (see Materials and Methods), namely “sex differentiation” (GO:0007548; padj = 5.35e-5; 47 genes) and “dosage compensation” (GO:0007549; padj = 2.61e-3; 15 genes) among those genes that were higher expressed at 96h AEL. None of the 165 terms was enriched in those genes higher at 72h AEL and among differentially expressed genes in the 96h vs. 120h comparison.

It has been shown that early eye-antennal disc stages are mainly characterized by patterning processes that subdivide the initially uniform disc into the individual organ anlagen [6,15,18,25,58–61]. Within organ-specific domains, further patterning processes define for instance the dorsal ventral axis in retinal field [62–64] or the proximal-distal axis of the antennae [65]. Additionally, the discs grow extensively throughout L1 and L2 stages mainly by cell proliferation [15,66]. Accordingly, the genes active at the late L2 stage were mostly involved in metabolic processes and generation of energy (Fig 1B). At the end of L2 stages, the patterning processes are mostly concluded and differentiation starts within each compartment. For instance, in the retinal field the progression of the differentiation wave is accompanied by a reduction in cell proliferation [8,10]. Therefore, mostly genes related to cell differentiation, nervous system development, pattern specification and compound eye development were significantly up-regulated at the mid L3 stage (Fig 1B). During the transition from the mid L3 stage to the late L3 stage again genes related to metabolism and energy production were down-regulated (Fig 1C). This may be explained by the fact that at 96h AEL the disc has not yet reached its final size, and cells anterior to the morphogenetic furrow still proliferate [10]. Also, directly behind the morphogenetic furrow one last synchronous cell division takes place to give rise to the last cells of the photoreceptor clusters (R1, R6 and R7) [10,60]. In the light of ongoing differentiation, genes active at the late L3 stage belonged to GO categories related to differentiation processes (Fig 1C). At this stage, terms related to processes taking place late during eye-antennal disc development [10] such as R7 cell differentiation or pigment metabolic process (Fig 1C) were also obtained.

In summary, the pairwise comparison of expression levels of genes expressed at the three studied stages of eye-antennal disc development recapitulate the key transition from pattering and proliferation processes to differentiation.

### Co-expression clustering reveals gene expression dynamics during eye-antennal disc development

**Fig 2:**
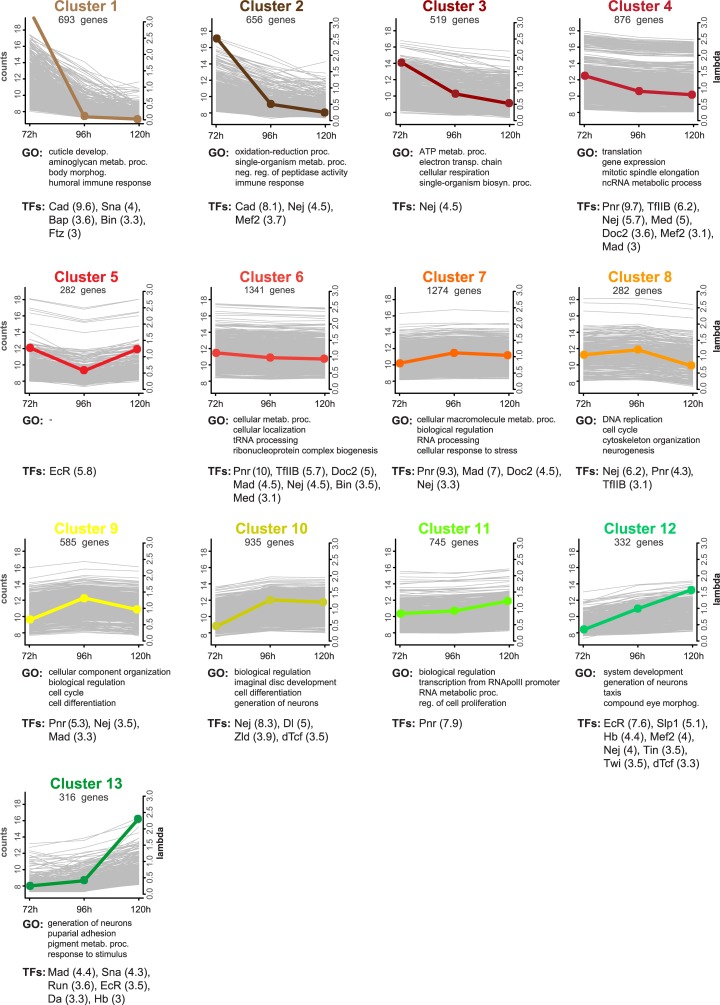
Co-expression clusters. All 13 profiles of co-expressed genes predicted by HTSCluster (see Materials and Methods). The number of genes assigned to a particular cluster are indicated below the cluster name. Colored dots represent relative expression levels (lambda value) of the genes of that cluster (y-axis on the right) at each stage. Background grey lines represent the normalized mean count of all genes belonging to a cluster (y-axis on the left). Below each cluster plot, the first four non-redundant GO terms enriched in the genes of that cluster are listed (see S1 Table) and the significantly enriched transcription factors (NES > 3, see S2 Table) are shown (the NES value is given in brackets).

In order to better characterize the different expression dynamics of the expressed genes, we performed a co-expression clustering analysis based on Poisson Mixture models [67]. Manual comparison of the different outputs showed that the 13 clusters predicted by one of the models (Djump, [68]) were non-redundant and sufficiently described all the expression profiles present in the data. A total of 8,836 genes could be confidently placed in one of these clusters (maximum a posteriori probability (MAP) > 99%). We ordered the predicted 13 clusters according to their expression profile (Fig 2): four clusters contained clearly early expressed genes, two of them contained genes expressed only at 72h AEL (cluster 1 and 2) and two contained genes predominantly expressed early, but also with low expression at 96h and/or 120h AEL (clusters 3 and 4); one cluster showed down-regulation at 96h AEL, but a peak of expression again at 120h AEL (cluster 5); the genes in the largest clusters showed almost constant expression throughout the three stages (clusters 6 and 7); one cluster showed constant expression at 72h AEL and 96h AEL and down-regulation at 120h AEL (cluster 8); one cluster showed a peak of expression at 96h AEL (cluster 9) and four clusters contained genes with predominantly late expression, one with high and constant expression at 96h AEL and 120h AEL (cluster 10), two with up-regulation in both transitions (cluster 11 and cluster 12) and one with genes expressed only at 120h AEL (cluster 13).

A GO enrichment analysis for the genes in the individual clusters showed that the genome-wide co-expression profiling and subsequent ordering of the clusters recapitulated the consecutive biological processes that take place during eye-antennal disc development with a great resolution (Fig 2, S1 Table). For instance, we found genes related to energy production mainly in clusters 2 and 3, while genes more specific for terms related to mitosis and cell cycle were found in clusters 4, 8 and 9. For example, cluster 8 grouped genes that were similarly high expressed at 72h and 96h AEL, and their expression decreases at 120h AEL. The enrichment of GO terms related to DNA replication and cell cycle control (Fig 2; S1 Table), corresponds with the fact that active proliferation takes place at these stages [60]. Thus, other genes that were grouped in this cluster, but for which no previous knowledge is available, are likely also related to these biological functions. Genes up-regulated in the later stages were separated in more specific clusters, and most of the enriched GO terms were related to differentiation and neuron and eye development. For instance, cluster 10 contained the more general term “imaginal disc development”, while cluster 12 showed enrichment for “compound eye morphogenesis”, and cluster 13 was the only with enriched terms related to pupation processes and pigmentation.

**Fig 3:**
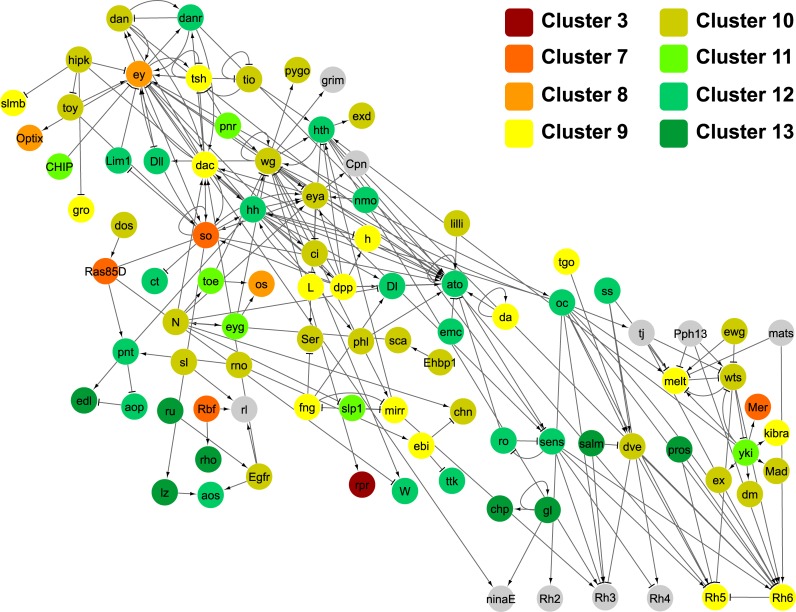
Assignment of key retinal genes to identified co-expression clusters. Directed gene interaction network (FlyOde,[50]) of the genes known to be involved in retinal development in *D*. *melanogaster*. Only genetic and protein-DNA interactions are included. The horizontal axis represents developmental time, with early L3 at the most left, and the vertical axis displays network hierarchy (please see [50] for details). Each node represents a gene, and these are color-coded according to the assigned co-expression cluster (see also Fig 2). In grey are genes that have not been allocated to any cluster, either because they did not pass the expression level threshold or the clustering confidence threshold (see Materials and Methods for details).

In order to get an overview of the presence of key eye developmental genes in the individual clusters, we mapped the clusters onto the FlyODE network (Fig 3) that comprises manually curated interactions of genes involved in retina development [50]. Only ten of the 146 genes of this network were not present in any of the 13 clusters, either because they did not pass the expression level or the cluster assignment thresholds (grey nodes in Fig 3). Apart from the cell death regulator *reaper (rpr)* (cluster 3), all other genes of this network are present in clusters 7 to 13 (Fig 3), that represent genes with constant expression throughout the three stages (clusters 7 to 9) or with increasing expression at later stages (clusters 10 to 13) (Fig 2). This observation is in accordance with the nature of the FlyODE network that covers mainly stages of retinal differentiation from the third instar to the adult [50]. The most prominent retinal determination genes [7,10] *eyeless (ey)*, *sine oculis (so)*, *dachshund (dac)* and *optix/six3* are present in clusters 7 to 9, while *eyes absent (eya)*, *twin of eyeless (toy)* and *eye gone (eyg)* were found in clusters 10 and 11. Among the genes expressed during late L3 stages in clusters 12 and 13 we found central genes involved in photoreceptor and neuronal differentiation [10] such as *atonal (ato)*, *glass (gl)* and *prospero (pros)* (Figs 2 and 3). Members of well-known developmental signaling pathways such as the Wnt, BMP and Hh pathways, EGFR, Notch and cell cycle related genes (e.g. *CycE*) were present in cluster 10 (Fig 3) that grouped genes with similarly high expression at 96h AEL and 120h AEL (Fig 2). Among genes which steadily increased in expression throughout the three studied stages (cluster 12), we found for instance *Delta* (*Dl*), which is one of the Notch receptor ligands [69] and has been shown to fulfill different roles during eye development [66,70,71]. Also, *anterior open* (*aop*) (also known as *yan*), a member of the JNK signaling pathway [72], which is described to repress photoreceptor differentiation [73] and also to determine R3 photoreceptor identity [74] was present in this cluster.

Although we sequenced the entire eye-antennal discs, we found many GO terms related to eye development with high enrichment scores, while very few GO terms specific for antenna and maxillary palps were observed (e.g. in cluster 12 “eye development” appears with *p* = 4.38e-24, “antennal development” with *p* = 4.37e-08 and no GO terms related to maxillary palps were found) (S1 Table). This finding may be a result of much more extensive research on eye specific developmental processes in comparison to the other organs that develop from the same imaginal disc. However, many GO terms related to leg formation and proximodistal pattern formation were highly enriched in the genes in cluster 9 (“proximaldistal pattern formation” with *p* = 4.48e-05), cluster 10 (“leg disc development” with *p* = 7.85e-20) and cluster 12 (“leg disc development” with *p* = 4.32e-12) (S1 Table). Since antennae and maxillary palps are serially homologue to thoracic appendages, pathways involved in leg, antenna and maxillary palp development are likely to share key regulators [17,65,75–80], suggesting that genes found in these clusters may also play a role in antenna or maxillary palp development.

The assignment of all expressed genes to their corresponding cluster is available along with the GEO submission number GSE94915 and on this website http://www.evolution.uni-goettingen.de/posnienlab/clusterSearch/search.html.

In summary, we showed that clusters with early expressed genes mainly represent metabolic and energy related processes, while clusters with late expressed genes represent more organ specific differentiation and morphogenetic processes. Therefore, we provide a dataset that contains the complete gene expression dynamics underlying the fundamental change from predominantly patterning and proliferation processes to the onset of differentiation from late L2 stages to late L3 larval instars [7,28].

### Transcription factors regulating *D*. *melanogaster* eye-antennal disc development

The co-expression of genes observed in the 13 clusters may be a result of co-regulation by the same transcription factors or combinations thereof. In order to test this hypothesis and to reveal potential central upstream regulators, we used the i-*cis*Target method [81] to search for enrichment of transcription factor binding sites in the regulatory regions of the genes within each of the 13 clusters (Fig 2, S2 Table). As basis for this enrichment analysis various experimental ChIP-chip and ChIP-seq datasets were used, namely those published by the modENCODE Consortium [82], the Berkeley *Drosophila* Transcription Network Project [83] and by the Furlong Lab [84,85].

One of the most noticeable results of the statistical ranking analysis was that genes in 9 of the 13 clusters showed significant enrichment for Nejire binding sites (Fig 2). Nejire (also known as CREB-binding protein (CBP)) is a zinc-finger DNA binding protein that functions as a co-activator that acts as bridge for other transcription factors to bind specific enhancer elements [86–88], which can explain why we find it to regulate such many target genes. Nejire/CBP has been shown to be involved in many processes during eye development and patterning in *D*. *melanogaster* [89,90] and mutations in this gene cause the Rubinstein-Taybi syndrome in humans [91] that among others is characterized by extensive problems during retinal development [92].

Similarly, Pannier was found enriched to regulate the genes of many clusters (clusters 2, 4, 6, 7, 8, 9 and 11) (Fig 2). This GATA transcription factor is involved in the establishment of the dorsal-ventral axis of the retinal field of the discs during early L1 and L2 stages [93,94], while later during L2 and L3 stages it is known to have a role in defining the head cuticle domain by repressing eye determination genes [64,94]. In both cases, Pannier is found in a very upstream position of the respective gene regulatory networks that define these cell fates [64,95].

Besides these highly abundant transcription factors, the clusters with genes predominantly expressed at later stages were also enriched for transcription factors already known to play a role in eye-antennal disc development. For instance, a significant number of Sloppy-paired 1 (Slp1) target genes are up-regulated at L3 stage (cluster 12, Fig 2) and this transcription factor is known to play a critical role in establishing dorsal-ventral patterning of the eye field in the eye-antennal disc [96]. A function of Daughterless (Da) (identified in cluster 13, Fig 2) is also described: it is expressed in the morphogenetic furrow, it interacts with Atonal and is necessary for proper photoreceptor differentiation [97]. Finally, Snail (enriched in cluster 1 and 13) and Twist (enriched in cluster 12) (Fig 2) were previously identified as possible repressors of the retinal determination gene *dac* [98] and our results indicate that they regulate also other genes during eye-antennal disc development.

Cluster 5 contained genes that show a peak in expression at 72h AEL and 120h AEL stages, which precede major stage transitions from L2 to L3 and from L3 to pupa stage, respectively. These transitions are characterized by ecdysone hormone pulses before larval molting and pupation [99]. The only potential transcription factor binding site that was significantly enriched was that of the Ecdysone Receptor (EcR), that has been shown to be expressed in the eye-antennal disc in the region of the progressing morphogenetic furrow [100].

Intriguingly, we did not find an enrichment for known transcription factors such as So or Ey. The most obvious explanation is that there is no ChIP-seq data available (e.g. for Ey) or that the available data for these factors (e.g. for So [48]) is not included as transcription factor binding information in the current i-*cis*Target database [81,101].

### Potential new regulators of eye-antennal disc development

The identification of well-known transcription factors suggests that the applied clustering approach indeed allows identifying key regulators of various processes taking place throughout eye-antennal disc development. Interestingly, we identified a few generally well-known upstream factors for which a potential role during eye-antennal disc development has not yet been described. For instance, in clusters of very early expressed genes, we found an enrichment of motifs for the transcription factor Caudal (Cad) (cluster 1 and 2) and the Hox protein Fushi tarazu (Ftz) (cluster 1) (Fig 2). Caudal is a downstream core promoter activator [102] and very recently it has been found that it cooperates with Nejire to promote the expression of the homeobox gene *fushi tarazu* (*ftz*) [103]. A Caudal-like transcription factor binding motif has been identified within Sine oculis (So) bound DNA fragments as identified by ChIP-seq [48], suggesting that So and Cad may co-regulate potential target genes in the eye-antennal disc.

The MADS-box transcription factor Myocyte enhancer factor 2 (DMef2) was predicted to regulate genes found in clusters 2, 4 and 12 (Fig 2). Using two independent *Dmef2*-Gal4 lines to drive GFP expression, we confirmed expression of *Dmef2* in lose cells attached to the developing eye-antennal discs (S2 Fig). Eventually, we found an enrichment of potential target genes of the C2H2 zinc-finger transcription factor Hunchback (Hb) in clusters 12 and 13, which are active mainly during mid and late L3 stages. Since GO terms enriched in these two clusters suggested an involvement in retinal development or neurogenesis (Fig 2, S1 Table), we examined a potential function of Hb in the eye-antennal disc in more detail.

### *hb* is expressed in retinal subperineurial glia cells

Using *in-situ* hybridization we found *hb* expression in two cell nuclei at the base of the optic stalk in the posterior region of late L3 eye-antennal discs (S3A Fig). With a Hb antibody we also detected the Hb protein in these two basally located nuclei (S3B and S3C Fig). DNA staining with DAPI showed that the Hb-positive nuclei are bigger than those of surrounding cells, suggesting that they are polyploid. Additionally, we tested two putative Gal4 driver lines obtained from the Vienna Tile library [104] (VT038544; S3D Fig and VT038545; S3E Fig). Both lines drove reporter gene expression in the two polyploid nuclei as described above. Note that both lines also drove the typical *hb* expression in the developing embryonic nervous system, but not the early anterior expression [105,106]. The regulatory region covered by the two Gal4 driver lines is located at the non-coding 3’ end of the *hb* locus accessible to DNA-binding proteins at embryonic stages 9 and 10 [83] (S4 Fig), a time when early-born neuroblasts express *hb* [55]. The lack of the early anterior expression may be explained by the fact that the DNA region covered by the driver lines does not seem to be bound by Bicoid during early embryonic stages [83] (S4 Fig). Based on these findings, we are confident that the regions covered by the two Gal4 driver lines (VT038544 and VT038545) recapitulate native *hb* expression.

**Fig 4:**
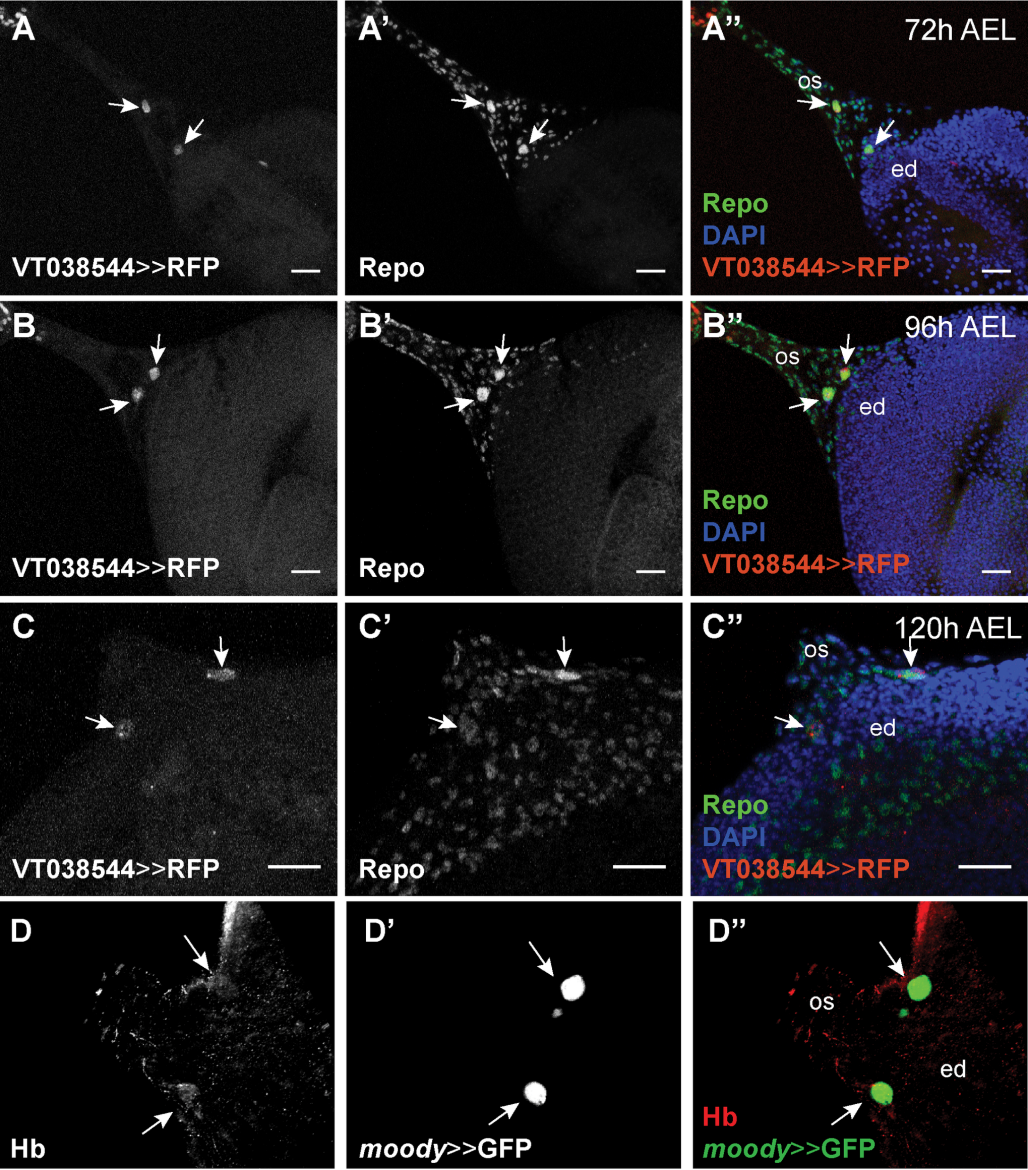
*hb* is expressed in migratory subperineurial glia cells. (A-C) *hb* expression (VT038544-Gal4 driving histone-bound RFP (UAS-H2B::RFP) (A-C), red in A”-C”) co-localizes (white arrows) with the pan-glial marker Repo (detected with rabbit α-Repo antibody (A’-C’), green in A”-C”) in two large cells. These cells migrate from the optic stalk during L2 stage (A”) into the posterior end of the eye-antennal disc at mid L3 stage (B”) and are located in the posterior region of the retinal field, at each side of the optic stalk, at late L3 stage (C”). (VT038544-Gal4 driver line obtained from the Vienna Tile collection, see S4 Fig for details) (D) Hb (detected with rabbit α-Hb antibody, red in D”) is present in the same cells (white arrows) that express the subperineurial glia cell marker *moody* (D’) (*moody*-Gal4 driving UAS-GFP, green in D”). In all pictures, anterior is to the right. Eye disc (ed), optic stalk (os). Scale bar = 20 μm.

The basal location of the *hb*-positive cells suggests that they may be retinal glia cells. Co-expression of *hb* with the pan-glial marker Repo (Fig 4A–4C) further supported this suggestion. Previous data has shown that two polyploid retinal subperineurial glia cells (also referred to as carpet cells) cover the posterior region of the eye-antennal disc [34,36]. In order to test, whether *hb* may be expressed in carpet cells, we first investigated the expression of the subperineurial glia marker Moody [107] and we found a clear co-localization with Hb (Fig 4D).

**Fig 5:**
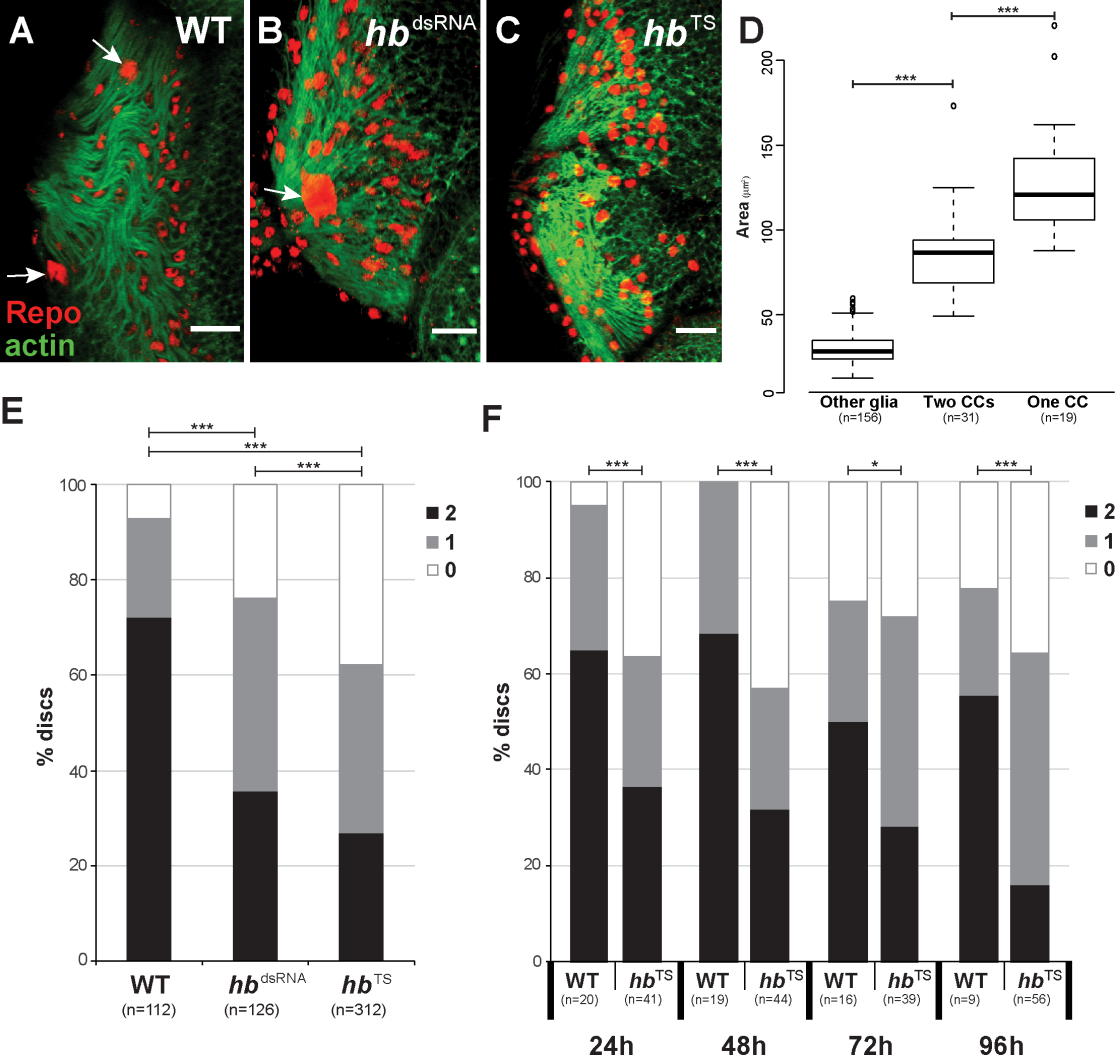
The number of polyploid glia cell nuclei is reduced after loss of Hb function. (A-C) Staining with rabbit α-Repo antibody (red) and Phalloidin (green) of late L3 eye-antennal discs in wild type (A), *repo* driven *hb* RNAi (B) and Hb temperature sensitive mutant (C). This figure represents the phenotypes that were analyzed in (D), where the number of polyploid nuclei (white arrows) have been quantified. In all pictures, anterior is to the right. Scale bar = 20 μm. (D) Box plot of glia cell nuclei size. When two carpet cells were present, these are significantly larger than other surrounding glia cells. When only one carpet cell was present in the retinal field, its nucleus was significantly larger than regular carpet cells. Significance calculated by t-test (“Two CCs” vs. “One CC”, homogeneous distribution of variances) and t-Welch-test (“Other glia” vs. “Two CCs”, not homogeneous distribution of variances); “***”: *p* < 0.0005. (E) Quantification of the number of polyploid nuclei observed in wild type (WT), *repo-*Gal4 and *moody-*Gal4 driven UAS-*hb* RNAi (*hb*^dsRNA^) and Hb temperature sensitive mutant (*hb*^*TS*^). (F) Quantification of the number of polyploid nuclei observed in late L3 eye-antennal discs of flies that were raised at 18°C until the indicated time points (24h AEL, 48h AEL, 72h AEL and 96h AEL), when they were transferred to the restrictive temperature of 28°C. In D and E, the black bar indicates percentage of discs with two polyploid glia cell nuclei, grey indicates discs with one polyploid glia cell nucleus and white indicates discs without polyploid glia cell nuclei. Pearson’s Chi-squared test was performed to determine if the distribution of the different number of cells (0, 1 or 2) was equal between wild type and RNAi. *: p-val < 0.05, ***: p-val < 0.0005.

Carpet cells migrate through the optic stalk into the eye-antennal disc during larval development [34,36]. Therefore, we followed the expression of the *hb* driver lines throughout late L2 and L3 larval stages (Fig 4A–4C). Already at the L2 stage, we could easily recognize the *hb*-positive cell nuclei by their large size (Fig 4A and 4A”; see also size quantification in Fig 5D). We could corroborate that these cells indeed migrated through the optic stalk during late L2 and early L3 stages (Fig 4A and 4B), and then entered the disc and remained basally in the posterior region of the disc, flanking the optic stalk (Fig 4C and 4C”). As previously observed for carpet cells [34], we never found *hb*-positive cell nuclei in the midline of the retinal field.

Taken together, these data show that *hb* is expressed in two polyploid retinal subperineurial glia cells (carpet cells) that enter the basal surface of the eye-antennal disc through the optic stalk during larval development.

### Hb function is necessary for the presence of polyploid carpet cells in the eye-antennal disc

The expression of *hb* in carpet cells suggested an involvement in their development. To test this hypothesis, we examined loss of Hb function phenotypes based on RNA interference (RNAi) driven specifically in subperineurial glia cells (*moody*-Gal4 driving UAS-*hb*^dsRNA^). Of four tested UAS-*hb*^dsRNA^ lines we used the most efficient line (see Materials and Methods) for the RNAi knock-down experiments. Additionally, we investigated eye-antennal discs of a temperature sensitive mutant (*hb*^*TS*^) [108]. Since Hb is necessary during embryogenesis [53,54], the analyzed flies were kept at 18°C during egg collection and throughout embryonic development, and they were only transferred to the restrictive temperature of 28°C at the L1 stage. We quantified the size differences between carpet cell nuclei and neighboring glia cells by measuring the nucleus area. The mean area of a Repo-positive glia cell was 30.42 μm^2^, while the carpet cell nuclei had a mean area of 86.10 μm^2^ (Fig 5A and 5D). Based on these clear results, the presence or absence of carpet cell nuclei could be confidently identified by α-Repo staining because of their significantly larger size (see Fig 5A and 5D).

The most common phenotype observed in late L3 eye-antennal discs of RNAi and mutant flies was the absence of one or both carpet cell nuclei (Fig 5A–5C). In wild type animals, we could unambiguously identify two carpet cell nuclei in 72% of the eye-antennal discs. In 21% of the analyzed discs, we found only one carpet cell nucleus (Fig 5E). In contrast, in 35% to 40% of the studied Hb loss of function discs only one carpet cell nucleus was observed (Fig 5B and 5E). This single polyploid Repo-positive nucleus was significantly larger than carpet cell nuclei in discs that contained two carpet cell nuclei (Fig 5D; 129.80 μm^2^ compared to 86.10 μm^2^ if two carpet cells were present). Additionally, the single carpet cell nuclei were mostly located in the midline of the retinal field (Fig 5B). No carpet cell nuclei could be observed in 24% and 38% of the eye-antennal discs originating from *moody*>>*hb*^dsRNA^ and *hb*^*TS*^ flies, respectively (Fig 5C and 5E). Note that we obtained comparable results when we expressed the *hb* dsRNA in all glia cells (*repo*>>*hb*^dsRNA^) or only in subperineurial glia cells (*moody*>>*hb*^dsRNA^).

To identify larval stages at which Hb function is crucial for carpet cell development, we transferred *hb*^*TS*^ flies to the restrictive temperature of 28°C at 24h AEL (early L1 stage), at 48h AEL (late L1), at 72h AEL (late L2) or 96h AEL (mid L3 stage) and assessed the presence of polyploid Repo-positive carpet cell nuclei in late L3 eye-antennal discs, respectively. In all cases, we found a significant reduction of the number of carpet cell nuclei when compared to control discs (Fig 5F). Although no clear significant differences in the number of carpet cells was detected between the consecutive experiments (S5 Fig), our results show that Hb function is necessary for the presence of polyploid carpet cell nuclei throughout larval development.

### Loss of Hb function affects carpet cell migration

**Fig 6:**
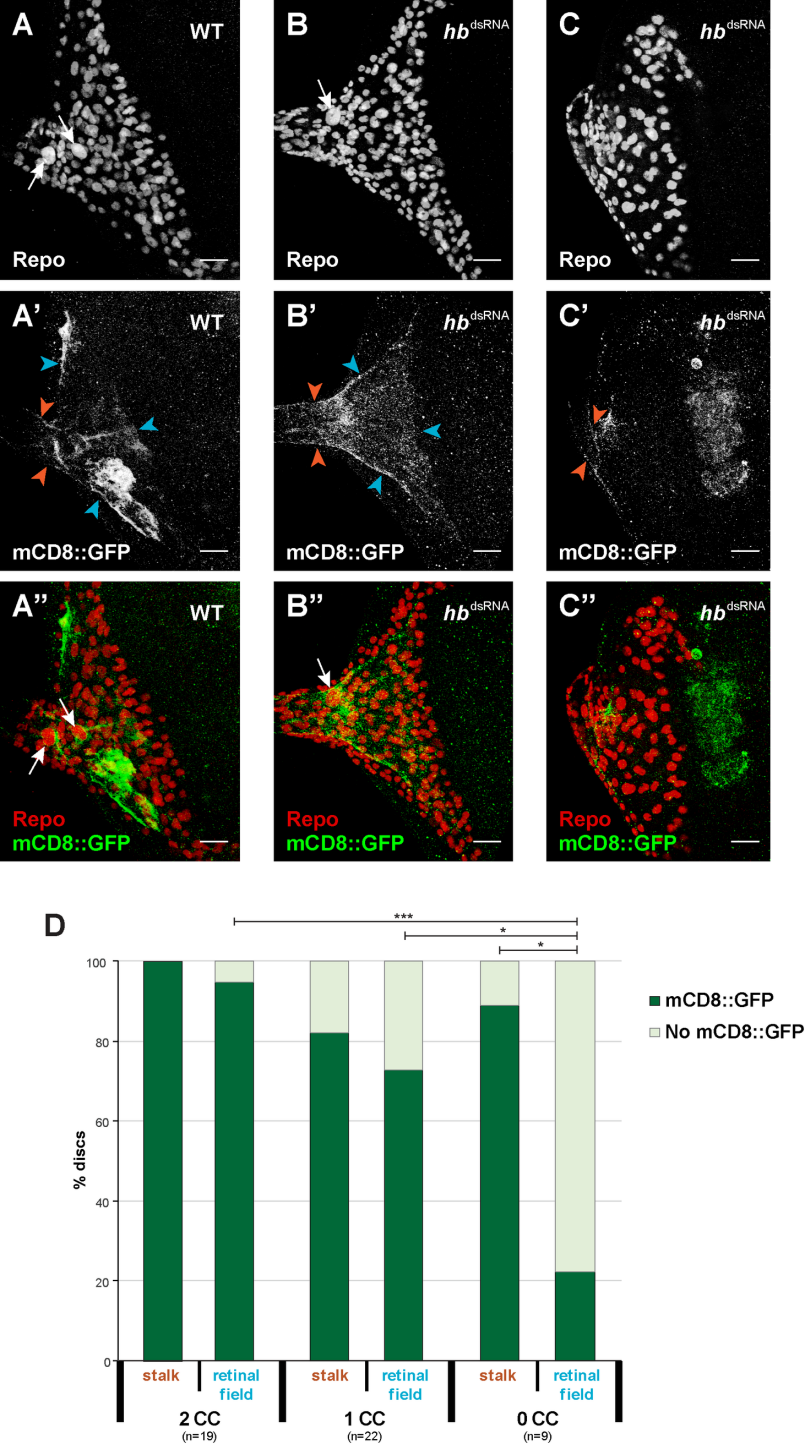
Carpet cell membranes after loss of carpet cells. (A-C) All glia cells are stained with rabbit α-Repo antibody (red in A”-C”). In control discs, two carpet cells can be identified by their large size (white arrows). After *moody* driven knock-down of *hb* expression (B and C) some discs present only one carpet cell nucleus (B, white arrow) and others no carpet cell nucleus (C) (refer to Fig 5 for percentages). (A’-C’) Membranes of carpet cells in late L3 eye-antennal discs are labelled with *moody-*Gal4 driven UAS-mCD8::GFP expression (green in A”-C”). The presence of these membranes was analyzed in the optic stalk (orange arrowheads) and in the retinal field (determined by the presence of these membranes in the posterior margins of the disc and their extension to the MF (blue arrowheads)). In all pictures, anterior is to the right. Eye disc (ed), optic stalk (os). Scale bar = 20 μm. (D) Quantification of the presence of carpet cell membrane as described in A’-C’. Pearson’s Chi-squared test was performed to determine significance between each pair of condition. *: p-val < 0.05, ***: p-val < 0.0005.

The observed loss of polyploid carpet cell nuclei could be a result of either the loss of the entire carpet cells, incomplete migration into the eye-antennal disc or loss of the polyploidy. To distinguish between these options, we tested whether also the carpet cell membranes were affected upon loss of *hb* expression, in addition to the polyploid nuclei. To this aim, we expressed *hb*^*dsRNA*^ specifically in subperineurial glia cells with a *moody*-Gal4 driver line together with a strong membrane marker (20xUAS-mCD8::GFP) to label the extensive carpet cell membranes (*moody*>>20xmCD8::GFP; *moody*>>*hb*^dsRNA^, Fig 6).

In the eye-antennal discs where two carpet cells could be identified, *moody* expressing membranes were always present in the optic stalk and in most cases (95%) they spanned the entire posterior region of the eye-antennal disc up to the morphogenetic furrow (Fig 6A and 6D). In discs with only one clear carpet cell nucleus, we observed a decrease both in the cases where mCD8::GFP was present in the optic stalk (82%) and even more in the retinal field (73%) (Fig 6B and 6D). Of the discs with no clear polyploid carpet cell nuclei (Fig 6C and 6D), also fewer (89%) showed *moody* expressing membranes in the stalk, although also the difference was not significant compared to controls. However, a very significant decrease in the percentage of discs with *moody* expressing membranes in the retinal field was observed in this case (22%).

### Loss of Hb function results in blood-brain barrier defects

Subperineurial glia cells cover the entire surface of the brain from larval stages onwards. They are an integral part of the protective blood-brain barrier by establishing intercellular septate junctions [109]. The blood-brain barrier prevents the substances that circulate in the hemolymph to enter the brain and helps maintaining the proper homeostatic conditions of the nervous system [110]. Since it has been shown that the carpet cells migrate through the optic stalk towards the brain during pupal stages [40], we tested, whether the loss of *hb* expression in developing carpet cells had an effect on the integrity of the blood-brain barrier.

**Fig 7:**
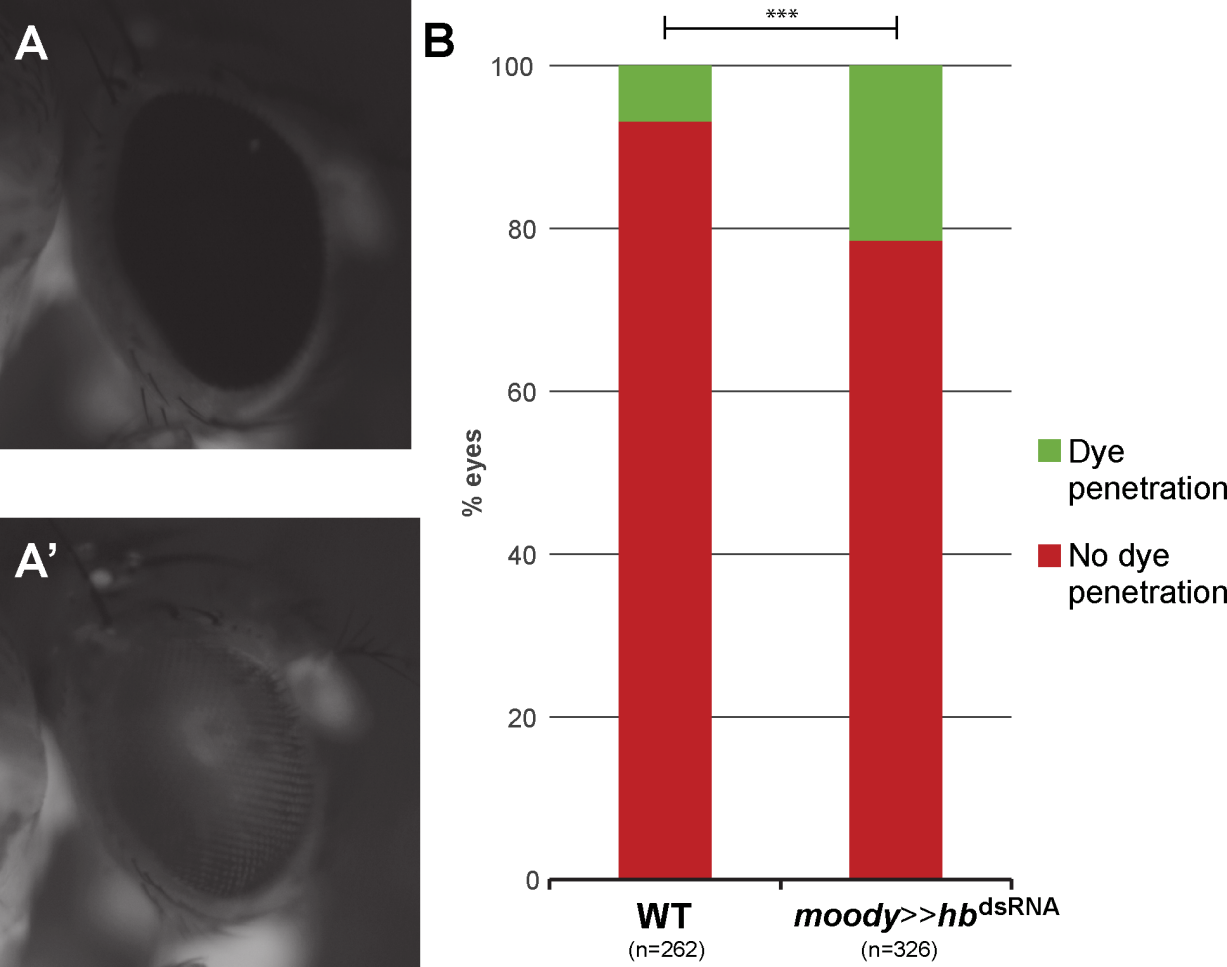
Blood-brain barrier function is impaired after loss of *hb* expression in carpet cells. (A) After injection of fluorescently labelled dextran into the abdomen of adult flies, animals with correctly formed blood-brain barrier show no fluorescence in the compound eye. (A’) In flies with incomplete blood-brain barrier, fluorescent dye can be observed in the compound eye as well as in the body. (B) Quantification of eyes with (green) or without (red) dye penetration. *hb* knock-down flies have a significant increase in the penetrance of dye into the eye, indicating a defective blood-eye barrier. Pearson’s Chi-squared test was performed to determine significance between the wild type results and the RNAi. ***: p-val < 0.0005.

To this aim, we injected fluorescently labeled dextran into the abdomen of *moody*>>*hb*^dsRNA^ adult flies and scored the presence of this dye in the retina of the flies. Animals with a properly formed blood-brain barrier showed a fluorescent signal in their body, but not in the retina (Fig 7A). However, in animals that had an incomplete blood-brain barrier, the dextran penetrated into the retina and fluorescence was observed in the compound eyes (Fig 7A’). Since it is known that blood-brain barrier permeability can increase after exposure to stress conditions [111,112], we only scored animals that survived 24h after the injection of dextran. In most cases, the two eyes of an individual presented different fluorescent intensities, and even no fluorescence in one eye but strong signal in the other. Therefore, we scored each eye separately. *moody*>>*hb*^dsRNA^ flies had a significantly higher rate of fluorescent retinas (*p* = 8.08e-7, χ^2^ test), indicating that their eyes were not properly isolated from the hemolymph circulating in the body cavity (Fig 7B).

In summary, our loss of function experiments further confirmed a central role of Hb in carpet cell development. Besides impaired retinal glia cell migration and axon guidance, we showed that upon loss of Hb function also the blood-brain barrier integrity is disrupted.

### Expression of putative Hb target genes in eye-antennal discs

**Fig 8:**
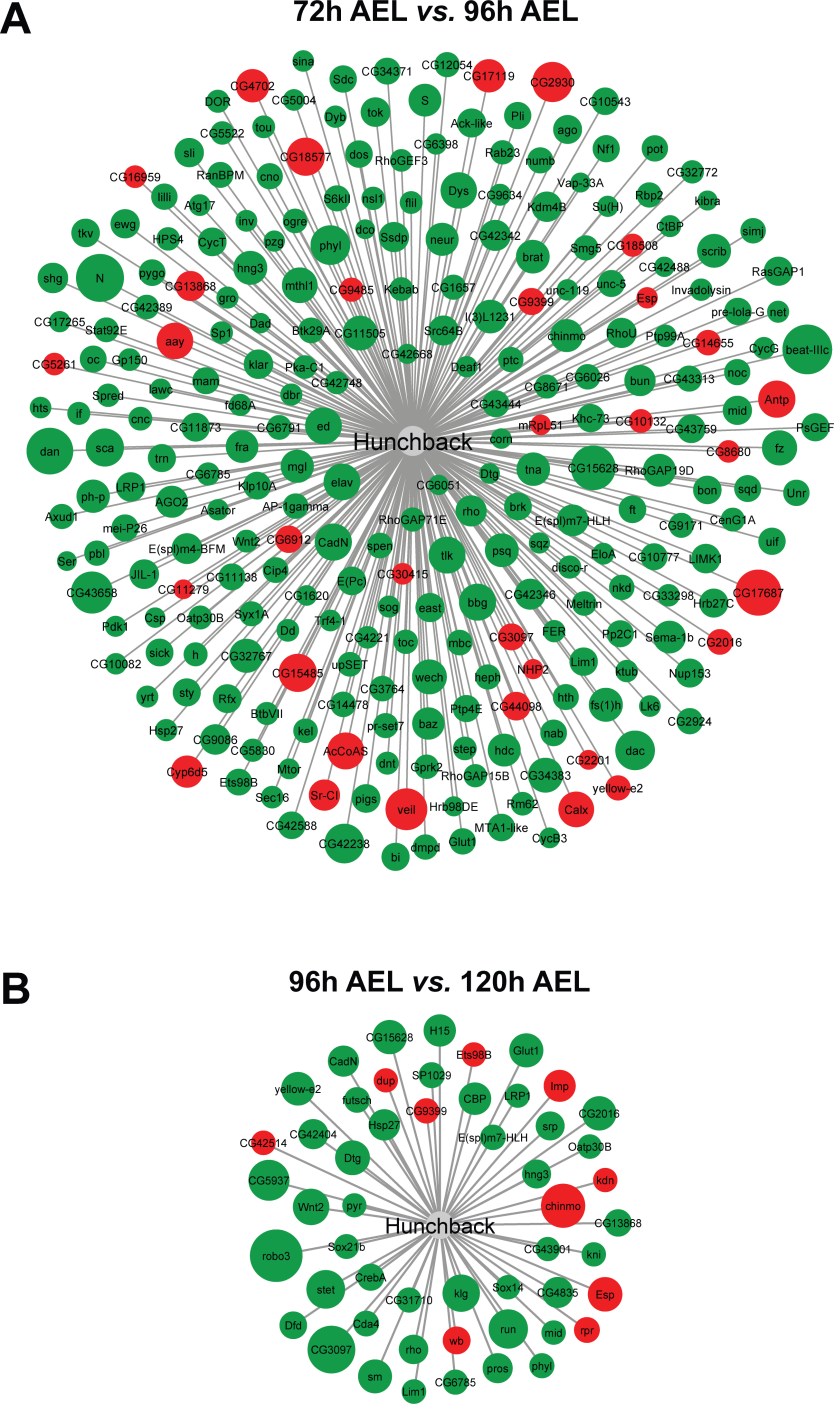
Differentially expressed putative Hb target genes. Green and red shaded circles are up- and down-regulated genes, respectively. Larger circle size indicates higher log2-fold change. (A) 267 genes from the high confidence list of Hb targets are differentially expressed in the eye-antennal discs during the transition from late L2 (72h AEL) to mid L3 (96h AEL) stages. 33 genes are down-regulated and 234 are up-regulated (see S4 Table). (B) 52 genes from the high confidence list of Hb targets are differentially expressed in the eye-antennal discs during the transition from mid L3 (96h AEL) to late L3 (120h AEL) stages. 10 genes are down-regulated and 42 are up-regulated (see S4 Table).

Since we have identified Hb because of an increase in expression of its target genes during 96h and 120h AEL stages and *hb* itself is only expressed in carpet cells, we also investigated, whether some of the targets were expressed in these cells. Using available ChIP-chip data for Hb from the Berkeley *Drosophila* Transcription Network Project (BDTNP) [83], we generated a high confidence list of 847 putative Hb target genes (see Materials and Methods for details), of which 585 were expressed in eye-antennal discs at least in one of the studied stages. More precisely, we found that 267 of these genes were differentially expressed in the transition from 72h to 96h AEL and only 52 were differentially expressed between 96h and 120h AEL (Fig 8, S4 Table). In both cases, most of these genes were up-regulated, suggesting that Hb mainly activates target gene expression in the eye-antennal disc. Focusing only on those target genes that resulted in the identification of Hb in our clustering approach (see above), we found that 77 of the 585 expressed putative Hb targets were present in clusters 12 and 13. We searched the GO terms for biological functions of these 77 genes and found that 17 code for transcription factors and up to 25 code for proteins integral to the cell membrane. A number of GO terms were related to neuronal development and eye development and to note is the presence of genes known to be related to glia cell migration and endoreduplication (S5 Table).

**Fig 9:**
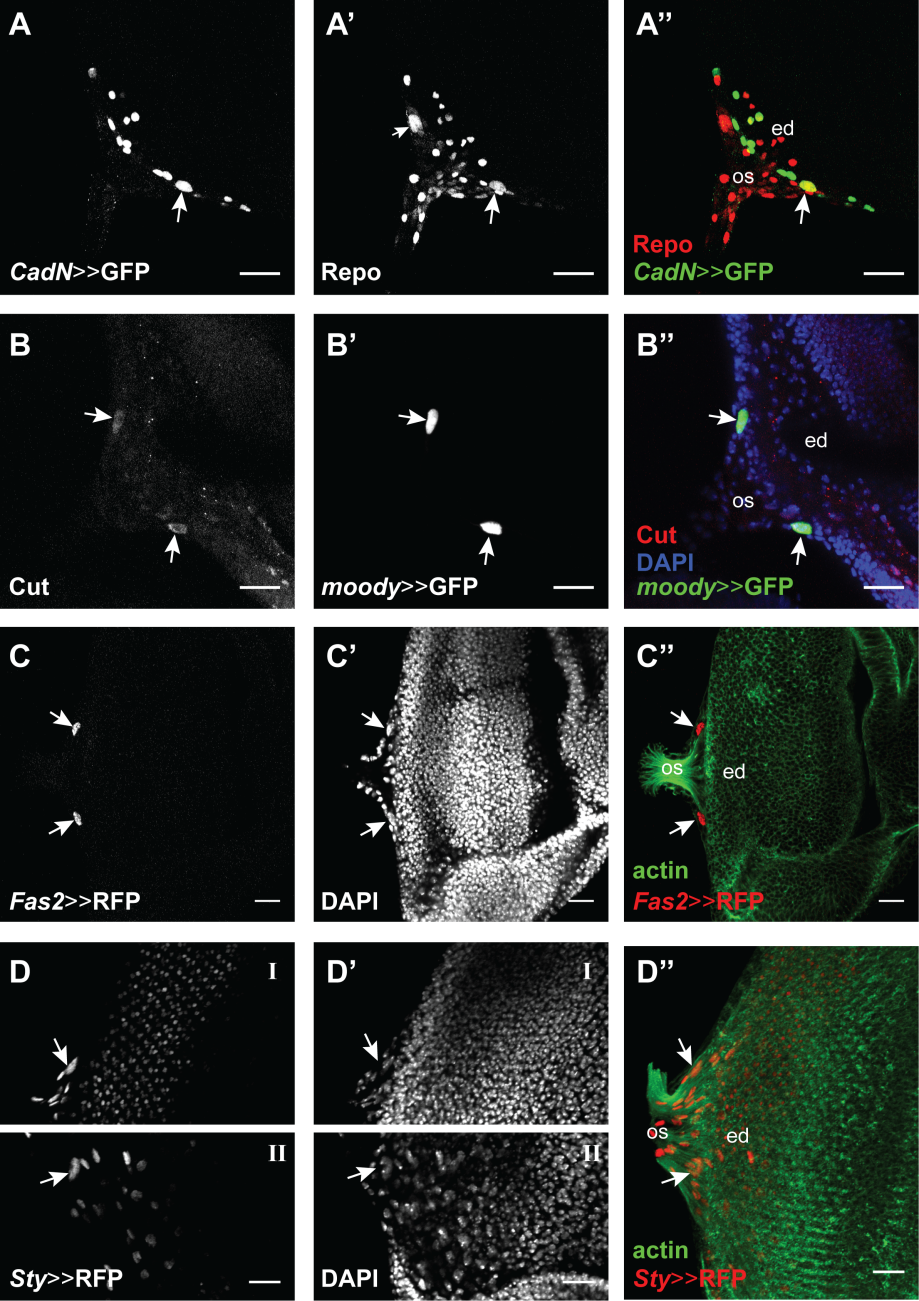
Expression of Hb target genes in the eye-antennal discs. Four of the tested target genes show expression in carpet cells. Eye disc (ed), optic stalk (os). Scale bar = 20 μm. (A) *CadN*-Gal4 drives UAS-GFP expression (green in A”) in one of the two carpet cells (white arrow), as well as other cells in the disc, possibly glia cells. (A’ and A”). Glia cells are marked with an antibody against the pan-glial marker Repo (rabbit α-Repo, red) and Repo-positive carpet cell nuclei are recognized by their large size (see also Fig 5D). (B) mouse α-Cut (red in B”) showed a clear signal in the two carpet cells (white arrows). (B’ and B”). Carpet cell nuclei were recognized by the expression of the subperineurial glia marker *moody* (*moody*-Gal4 driving UAS-GFP expression, green). DAPI shows the eye-antennal disc surface. (C) *Fas2*-Gal4 drives UAS-H2B::RFP (red in C”) expression in the two carpet cells (white arrows). (C’ and C”) Carpet cell nuclei are recognized by their large size with DAPI and their location on the posterior edge of the retinal field between the outgoing axons visualized with Phalloidin staining (green). (D) *Sty*-Gal4 drives UAS-H2B::RFP (red in D”) expression in the two carpet cells (white arrows), as well as in other cells in the disc. Due to folding of the imaged disc, the right (D-I) and left (D-II) carpet cells where not found in the same focal plane. (D’ and D”) Carpet cell nuclei are recognized by their large size with DAPI and their location on the posterior edge of the retinal field between the outgoing axons visualized with Phalloidin staining (green).

Based on their annotated GO terms, predicted or known cellular location and the availability of driver lines and antibodies, we selected 13 of these target genes and tested if they were expressed in carpet cells at 120h AEL. For 8 out of the 13 selected targets we found no clear expression related to carpet cells (*archipelago* (*ago*), *Delta* (*Dl*), *knirps* (*kni*), *rhomboid* (*rho*), *roundabout 3* (*robo3*), *Sox21b*, *Src oncogene at 64B* (*Src64B*) and *thickveins* (*tkv*)). This could be because they were false positives, but they could also be expressed at earlier stages than analyzed here or the used driver constructs did not include the regulatory regions to drive expression in carpet cells. Tkv for instance could still be an interesting candidate because it has been implicated in retinal glia cell development [113]. *brinker* (*brk*), *Cadherin-N* (*CadN*), *cut* (*ct*), *Fasciclin 2* (Fas2) and *sprouty* (*sty*) showed expression in carpet cells (Fig 9). *brinker* (*brk*) was very broadly expressed in the eye-antennal disc (S6C and S6D Fig). Although we could only observe expression in one of the two cells in every eye-antennal disc we analyzed, *CadN* is clearly expressed in carpet cells (Fig 9A). Recent data demonstrated that CadN, a Ca^+^ dependent cell adhesion molecule, is necessary for the proper collective migration of glia cells [114], a key feature of carpet cells. As it has previously been published, Cut is expressed in subperineurial glia cells (Fig 9B) [115]. The Cut protein is present in carpet cells already at L2 stage and remains until late L3 stage (Fig 9B). It has been shown that Cut is necessary for proper wrapping glia differentiation (note that the wrapping glia expression is found in another focal plane in Fig 9B) and to correctly form the large membrane processes that these cells form [115]. Interestingly, carpet cells have a similar morphology, with very large membrane surface and extensive processes that reach to the edge of the retinal field. In contrast, retinal perineurial glia do not have this morphology and do not express Cut. Also, *Fas2* (Fig 9C) and *sty* (Fig 9D) were clearly expressed in carpet cells as well as in several other cells in the eye-antennal disc. Sty and Fas2 are negative regulators of the EGFR signaling pathway that is involved in retinal glia cell development and photoreceptor differentiation [116–121].

Please note that we used antibodies to detect target genes if they were available. Given the nature of the CadN and Fas2 proteins [122,123], we detected broad membrane associated expression for both proteins (S6 Fig). Therefore, we decided to study the expression of those two candidates using available Gal4 lines (see above and Fig 9A and 9C). For both lines, we found at least one high confidence Hb motif (S6 Table).

In summary, we showed that 5 of the 13 computationally predicted Hb target genes that we tested were expressed in carpet cells, suggesting that our bioinformatic pipeline allows the identification of new potential regulators of carpet cell development.

## Discussion

### Genome wide expression dynamics allows the identification of known and new transcription factors involved in eye-antennal disc development

Although compound eye development is one of the most extensively studied processes in *D*. *melanogaster*, a comprehensive understanding of genome wide gene expression dynamics is still missing. A previous genome wide expression study based on Microarray data has shown that between 5,600 and 6,100 genes are active in the retinal part of the eye-antennal disc at the late L3 instar stage [49]. Given that we studied the entire eye-antennal discs at three time points, our observation that 9,194 of all annotated *D*. *melanogaster* genes are expressed in at least one stage is likely to provide a meaningful estimate of the number of active genes throughout eye-antennal disc development. The pairwise comparison of the studied stages (Fig 1), as well as the more defined clustering approach (Fig 2), revealed an extensive remodeling of the transcriptomic landscape during eye-antennal disc development.

It has previously been shown that co-expression of genes is likely to be a result of regulation by similar or even the same transcription factors [124–128]. This basic assumption has been successfully used to identify central transcriptional regulators in developmental gene regulatory networks [44,85,129–133]. Here, we identified several transcriptional regulators, such as Pnr, Nejire, Slp1, Da, Snail, Twist and EcR (Fig 2) that have previously been shown to regulate various aspects of eye-antennal disc development, but we also found new transcriptional regulators. For instance, the MADS-box transcription factor Myocyte enhancer factor 2 (DMef2) was predicted to regulate a number of genes in various clusters. The detection of *Dmef2* expression in lose cells attached to the developing eye-antennal discs (S2 Fig) confirmed that our result is not an artefact, but rather specific. Since, DMef2 is crucial for the development of muscle and heart tissue [134] these cells could be precursors of future head muscles. However, some recent findings could also hint towards an important role of this transcription factor in eye development since DMef2 has been implicated in circadian behavior [135] and larval eye and adult ocelli function [136]. These findings certainly encourage additional research on the possible role of DMef2 in photoreceptor cell development.

Taken together, the combination of dynamic gene expression clustering and upstream factor enrichment provides an excellent basis for the identification of potential new regulators involved in a given biological process.

### Integration of genomic resources: Opportunities and limitations

We identified a number of putative direct transcriptional regulators, although the ChIP-seq experiments that identified the direct interaction of a transcription factor with its target genes were not specifically performed in eye-antennal disc tissue at the stages we studied here. Indeed, most data available in current databases is based on experiments in embryonic or adult stages [82,83,85,137,138]. Interestingly, the enrichment of Caudal in clusters 1 and 2 (S2 Table) is based on data from a ChIP-seq experiment performed in adult flies [82], but does not represent an experiment performed in embryos [83]. This could indicate that Caudal has very different downstream targets during embryogenesis compared to its target genes at later stages. Although this observation may also indicate that the parameters and thresholds used in the different ChIP-seq experiments are very different, a large degree of tissue and stage specific target genes is expected. In the light of this specificity, we may miss eye-antennal disc specific target genes in our survey, but we are confident that one can identify a representative set of target genes to justify further tissue and stage specific ChIP-seq experiments if necessary.

We failed to identify enriched motifs for classical retinal determination genes such as So, Ey or Glass (Gl) in any of the 13 clusters, due to the fact that respective ChIP-seq data is either not available or not included in the i-*cis*Target database. Therefore, our analysis is biased towards those transcription factors for which ChIP-seq data is publicly available. We performed a preliminary power analysis to evaluate the potential to pick up an enrichment of So binding sites. We identified those clusters that contained 112 previously identified putative So/Eya target genes [47] and we found 76 of them distributed throughout all clusters (S3 Table). The most representative clusters were cluster 2 with 17 (of 656 genes in the cluster) and cluster 10 with 15 (of 935 genes in the cluster) So/Eya target genes. Given that the significantly enriched transcription factors with relatively low NES scores of 3.7 (Mef2 in cluster 2) and 3.5 (dTcf in cluster 10) (Fig 2) are predicted to regulate 57 and 559 target genes, respectively, this analysis very likely would not provide enough power to pick up a statistically significant enrichment. However, we detected an enrichment for Nejire although its putative target genes are present in 9 of 13 clusters. One explanation could be that the Nejire protein is detected ubiquitously in the entire eye disc [90] where it acts as a co-regulator [86–88] and thus influences the expression of many target genes. In contrast, classical retinal determination genes are dynamically expressed in more restricted domains, what is recapitulated by a highly complex regulation of these genes [139,140] and many different functions throughout eye-antennal disc development. Previous studies aiming at the identification of target genes resulted in limited lists of putative target genes for So (112 high confidence targets [47]) and Ey (20 direct targets in the retinal part of the eye-antennal disc [46]) suggesting that retinal determination genes regulate a restricted number of genes in a highly context dependent manner.

### A new role of Hb in retinal glia development

The combination of our genome wide expression analysis with functional experiments revealed a novel role of the C2H2 zinc-finger transcription factor Hunchback (Hb) in carpet cell development. Carpet cells have been shown to be a sub-population of the subperineurial glia cells due to shared key cellular features, such as the formation of extensive septate junctions [34]. However, a well-established subperineurial glia cell driver (NP2276 [141]) does drive reporter gene expression in brain subperineurial glia, but we could not detect expression in carpet cells. In contrast, we only detected *hb* expression in carpet cells and not in any subperineurial glia cells in the larval brain (results confirmed both using immunostaining and two driver lines (VT038544 and VT038545)). Additionally, if we compare our list of putative Hb target genes with the 50 genes enriched in adult blood-brain barrier surface glia [51], we only find *Fas2* to be present in both datasets. All these data suggest that carpet cells are indeed a retina specific subperineurial glia cell type that is molecularly very distinct from brain subperineurial glia cells.

The carpet cells function as a scaffold for undifferentiated retinal perineurial glia cells, which migrate into the disc to find the nascent axons of differentiating photoreceptors [34] and to guide them through the optic stalk [35]. In accordance with these roles, our Hb target gene analysis revealed many candidate target genes with GO terms related to axon guidance (S5 Table). Upon loss of Hb function, the most obvious phenotype was the lack of polyploid cell nuclei and carpet cell membranes in the eye-antennal discs. In contrast to our results, previous studies have shown that carpet cell ablation or a reduction of their size causes over migration of perineurial glia cells anterior to the morphogenetic furrow [34,113]. The corresponding experiments are based on the induction of cell death in *moody* expressing cells [34] and thus affect not only the carpet cells, but also for instance all other subperineurial glia of the brain. Since *hb* is specifically expressed carpet cells, the phenotype obtained here may be more specific. It remains to be studied, however, how carpet cells and subperineurial glia cells of the brain may interact to regulate perineurial glia cell migration in the eye-antennal discs.

Carpet cells possess a high level of plasticity since the loss of one carpet cell from the retinal field resulted in one larger carpet cell (i.e. larger polyploid nucleus) located in the midline of the eye field. In these cases, also no perineurial glia cell over migration could be observed and the membrane of this single cell seemed to sufficiently cover the full retinal field indicating that a single carpet cell could compensate the loss of the other one.

### Carpet cells are an integral part of the blood-brain barrier

The loss of *hb* expression affected the integrity of the blood-eye barrier, a subset of the blood-brain barrier (Fig 7). This phenotype was not as striking as previously published for *moody* mutant flies [39], where all surface glia cells were affected, suggesting that the carpet cells may indeed only contribute to the retinal part of the blood-brain barrier (i.e. the blood-eye barrier). Additionally, in our analysis of the *moody* positive cell membranes present in the optic stalk in *hb*^*RNAi*^ flies we found still carpet cell membranes in the optic stalk in up to 80% of the knock-down discs (Fig 6D). This is a similar proportion as we find in our blood-brain barrier assay (Fig 7).

The largest portion of the blood-brain barrier is already established by the end of embryogenesis [142,143], while the eye-antennal disc and developing photoreceptors seem to be accessible for the hemolymph during larval and very early pupal stages. Indeed, the final closure of the blood-brain barrier in the region where the optic stalk contacts the brain (i.e. the blood-eye barrier) is only established late during pupal development [109,144]. The rather late formation of the blood-eye barrier may be related to the dual role of carpet cells, which first migrate into the eye-antennal disc and only during pupal stages migrate back into the optic stalk towards the brain lobes. By mid-pupa stages they are located at the base of the brain lamina [40], where they remain throughout adult life and form septate junctions that isolate the brain and retina from the hemolymph [109]. The use of the newly analyzed driver lines, which drive expression specifically in the carpet cells represent an excellent starting point to study the migration of these cells throughout late larval and pupae stages in more detail.

### Implications on the role of Hb and the origin of carpet cells

The lack of ployploid large carpet cells during larval stages and the loss of blood-brain barrier integrity could either indicate a central role of Hb in specifying carpet cell identity entirely or a more specific role in defining aspects of carpet cell identity such as polyploidy and/or its migratory behavior. Based on our data, we propose the following cellular functions:

First, the lack of polyploid nuclei could hint towards a role of Hb in regulating the extensive endoreplication process necessary to generate such huge cell nuclei. Indeed, in our target gene analysis we found *archipelago (ago)* as one potential target. Ago has been shown to induce degradation of CyclinE (CycE) [145], a crucial prerequisite for efficient endoreplication cycles [146]. Second, Hb could be involved in establishing the migratory behavior of carpet cells. In the list of putative Hb target genes, we found many genes with GO terms related to cell migration and, some even specifically with the “glia cell migration” term. Additionally, many of the identified Hb target genes are involved in the epidermal growth factor (EGF) pathway that has various roles in development and cancer [147–149] including cell division, differentiation, cell survival and migration [150,151]. The list of Hb target genes up-regulated at 96h and 120h AEL in eye-antennal disc development includes both positive (*rhomboid*, *Star* and *CBP*) and negative regulators (*Fasciclin2* and *sprouty*) of this pathway. *Fas2* and *sprouty* are specifically expressed in carpet cells (Fig 9C and 9D), suggesting that Hb may actively influence the migratory behavior of carpet cells by activating genes involved in EGFR signaling regulation. Another putative target gene of Hb is *Ets98b* (Fig 8B), the ortholog of which has recently been shown to induce ectopic cell migration upon misexpression during early embryonic development in the common house spider *Parasteatoda tepidariorum* [152]. Finally, Nejire has been shown to be involved in glia cell migration in the peripheral nervous system in *Drosophila* [153], and we found it as putative regulator of genes in cluster 13. It remains to be established, whether Nejire and Hb may collaborate during carpet cell development.

Although carpet cells fulfill fundamental functions, it is still unclear where these cells originate from. Based on observations by Choi and Benzer (1994) using the enhancer trap line M1-126, these cells originate in the optic stalk where they are present at late L2 stage [36]. It has also been proposed that carpet cells may originate from a pool of neuroblasts in the neuroectoderm during embryogenesis [154] or in the optic lobes [155]. A clonal analysis using the FLP-out system suggests that various retinal glia cell types, including the carpet cells, originate from at least one mother cell at L1 larval stage [35]. Since only one polyploid cell nucleus seems to originate from one clone [35] and we show that in some loss of Hb imaginal discs only one polyploid cell nucleus is present, we propose that the two carpet cells may originate independently probably from two mother cells defined during L1 stages. Our observation that loss of Hb function resulted in loss of polyploid carpet cell nuclei when *hb*^*TS*^ mutant flies were transferred to the restrictive temperature during the L1 larval stage, further supports an involvement of Hb during this stage (Fig 5 and S5 Fig).

The exact role of Hb, however, is still unclear and will require further in-depth analyses. The newly analyzed driver lines in combination with the extensive list of potential Hb target genes, of which many are likely to be expressed in this specific glia cell type, represents a valuable resource to address the questions concerning the origin of these cells in more detail.

### Conclusions

The identification of Hb has only been possible because we studied the dynamic expression profiles of all genes expressed during eye-antennal disc development. Since the RNA levels of *hb* in the entire eye-antennal disc are negligible, we could identify Hb as central factor only through the expression profiles of its putative target genes, which are steadily up-regulated throughout development. This up-regulation is very likely due to the large cell bodies of the carpet cells, which need to produce high amounts of cytosolic or membrane bound proteins. At earlier stages, carpet cells are not yet in the eye-antennal discs, and *hb* expression could only been have identified by studies focused on the optic stalk. Moreover, we could show that refining the putative Hb target genes by incorporating the expression data results in a list that contains genes with GO terms highly specific for the putative function of Hb in carpet cells. Following the stepwise identification of putative target genes, we could confirm a high number of those experimentally.

All these findings demonstrate that the combination of high throughput transcript sequencing with a ChIP-seq data based transcription factor enrichment analysis can reveal previously unknown factors and their target genes, and therefore increase the number of connections within the underlying developmental GRNs. Other studies have searched for regulating transcription factors that were in the same co-expression clusters as its targets genes [44]. However, upstream orchestrators do not necessarily have the same expression levels as their targets. Therefore, the combination of ChIP-seq methods with RNA-seq co-expression analyses has proven to be a powerful tool to identify new developmental regulators that can complement other studies based on reverse genetics.

## Materials and methods

### RNA extraction and sequencing

*D*. *melanogaster* (OregonR) flies were raised at 25°C and 12h:12h dark:light cycle for at least two generations and their eggs were collected in 1h windows. Freshly hatched L1 larvae were transferred into fresh vials in density-controlled conditions (30 freshly hatched L1 larvae per vial). At the required time point, eye-antennal discs of either only female larvae (96h and 120h AEL) or male and female larvae (72h AEL) were dissected and stored in RNALater (Qiagen, Venlo, Netherlands). Please note that a mix of males and females was dissected for the 72h AEL samples because it is not possible to morphologically distinguish the sex at this stage. 40–50 discs were dissected for the 120h samples, 80–90 discs for the 96h samples and 120–130 discs for the 72h samples. Three biological replicates were generated for each sample type.

Total RNA was isolated using the Trizol (Invitrogen, Thermo Fisher Scientific, Waltham, Massachusetts, USA) method according to the manufacturer’s recommendations and the samples were DNAseI (Sigma, St. Louis, Missouri, USA) treated in order to remove DNA contamination. RNA quality was determined using the Agilent 2100 Bioanalyzer (Agilent Technologies, Santa Clara, CA, USA) microfluidic electrophoresis. Only samples with comparable RNA integrity numbers were selected for sequencing.

Library preparation for RNA-seq was performed using the TruSeq RNA Sample Preparation Kit (Illumina, catalog ID RS-122-2002) starting from 500 ng of total RNA. Accurate quantitation of cDNA libraries was performed using the QuantiFluordsDNA System (Promega, Madison, Wisconsin, USA). The size range of final cDNA libraries was determined applying the DNA 1000 chip on the Bioanalyzer 2100 from Agilent (280 bp). cDNA libraries were amplified and sequenced (50 bp single-end reads) using cBot and HiSeq 2000 (Illumina). Sequence images were transformed to bcl files using the software BaseCaller (Illumina). The bcl files were demultiplexed to fastq files with CASAVA (version 1.8.2).

### Bioinformatics analysis

#### Quality control

Quality control was carried out using FastQC software (version 0.10.1, Babraham Bioinformatics). All samples but one (“72hC” sample) had quality score >Q28 for all read positions. 12% of reads in sample “72hC” had an “N” in position 45, probably due to an air bubble in the sequencer. Following recently published guidelines [156,157], sequences were not trimmed but the aligner software was used to this purpose instead with very stringent parameters (see below). All raw fastq files are available through GEO Series accession number GSE94915.

#### Read mapping

Transcript sequences (only CDS) of *D*. *melanogaster* (r5.55) were downloaded from FlyBase and only the longest transcript per gene was used as reference to map the reads using Bowtie2 [158] with parameters–very-sensitive-local–N 1. The number of reads mapping to each transcript were summarized using the command idxstats from SAMtools v0.1.19 [159]. A summary of raw read counts mapped to each gene and time point is available at the GEO repository (GSE94915).

#### Pairwise comparison of gene expression and GO annotation

For each pair-wise comparison (72h AEL vs. 96h AEL and 96h AEL vs. 120h AEL) HTSFilter [160] was used with default parameters to filter out genes with very low expression in all samples. For the remaining genes in each pair-wise comparison, differential expression was calculated using DESeq2 v1.2.7. with default parameters [161]. Gene Ontology (GO) terms enrichment analysis for Biological Process was performed with GO TermFinder [162] with default parameters. Only the first non-redundant terms were plotted.

To gain some superficial estimate of the number of differentially expressed genes between 72h AEL and 96h AEL that may be a result of sex differences (note that a mix of both sexes is present at 72h, while only females were sequenced at 96h) we extracted a list of 165 GO terms associated with the search term “sex” from the gene ontology website (http://www.geneontology.org/) and searched for an overlap with enriched GO terms in the pairwise stage comparisons.

#### Gene expression clustering

HTSFilter [160] was used with default parameters to discard lowly expressed genes across all samples. The function PoisMixClusWrapper from the library HTSCluster [67] was applied on the rest of genes with the parameters: gmin = 1, gmax = 25, lib.type =“DESeq”.

Different model selection approaches are used by HTSCluster (i.e. to identify the number of clusters that best describe the data (see [67]). Our previous experience with this package had shown that the BIC and ICL methods report always as many clusters as we have allowed to test for (corresponding to the “gmax” parameter). Also for this analysis, both methods reported 25 as the most likely number of clusters, which was the input “gmax” value. Consequently, we discarded these results and we only analyzed the results of the methods that use slope heuristics to calculate the best number of clusters, namely DDSE and Djump. The DDSE method reported 19 clusters, with 8,626 genes having MAP > 99% while the Djump method reported 13 clusters, with 8,836 genes having MAP > 99%. Careful inspection of the lambda values of each of these clusters showed that the additional clusters predicted by the DDSE method presented negligible variation to the 13 clusters predicted by Djump. Additionally, GO term analysis (see below) of the genes in the 19 clusters predicted by DDSE showed redundant terms for the very similar additional clusters, which was not the case with the 13 clusters predicted by Djump. Therefore, we concluded that the additional clusters present in the DDSE prediction were unlikely to represent significant biological differences and that the 13 clusters predicted by Djump could sufficiently describe the profiles of the groups of co-expressed genes and we used them for all following analyses.

Genes with predicted MAP < 99% were discarded. Cluster assignment results can be found at the GEO repository (GSE94915) and easily accessible here: http://www.evolution.uni-goettingen.de/posnienlab/clusterSearch/search.html. For the plots, the variance stabilizing transformation from DESeq2 [163] library was used to normalize the background read count of the genes belonging to each cluster.

The Gene Ontology terms enriched in each cluster of genes were obtained with the plugin BiNGO [164] in Cytoscape v3.1.1 [165] with default parameters. The ontology terms and corresponding *D*. *melanogaster* annotations were downloaded from geneontology.org [166,167] (as of January 2015).

The transcription factors enriched to regulate the genes of each cluster were obtained with the i-*cis*Target method [81] with the following parameters: dm3 assembly, only “TF binding sites”, 5 Kb upstream and full transcript as mapping region, 0.4 as minimum fraction of overlap, 3.0 as NES threshold and 0.01 ROC threshold.

#### Identification of Hb target genes

The i-*cis*Target method [81] to detect transcription factor enrichment in the regulatory regions of co-regulated genes is based on the arbitrary partition of the *D*. *melanogaster* genome in more than 13,000 regions. All genes included in a particular region are associated to the transcription factor binding interval, resulting maybe in an unspecific association between transcription factor and target genes. Therefore, we aimed to generate a more confident list of putative Hb target genes in the eye-antennal disc. From Berkeley *Drosophila* Transcription Network Project (BDTNP) site [83], BED files were downloaded for the Hb (anti-Hb (antibody 2), stage 9) ChIP-chip experiment (Symmetric-null test and 1% FDR cutoff). The LiftOver tool from UCSC Browser [168] was used to transform the dm2 coordinates into the dm3 assembly. The closest gene to each ChIP-chip interval was identified with the script annotatePeaks.pl from the HOMER suite of tools [169]. Enrichment for the Hb motif in the regulatory regions of the identified genes were confirmed with the script findMotifGenome.pl from the same suite. The identified enriched Hb motif (as matrix) was used to search again the closest genes to the ChIP-chip intervals using the script annotatePeaks.pl with the parameters tss–size -1000,1000 –m *motif_matrix*. The genes with at least one instance of the motif were selected as Hb high confident targets. Cytoscape v3.1.1 [165] was used to visualize high confidence Hb targets which are significantly up- and down-regulated in the 72h AEL to 96h AEL and 96h AEL to 120h AEL transitions.

### Experimental procedure

#### Fly lines and crosses

The following fly lines were used: UAS-*hb*^dsRNA^ (Bloomington Stock Center #54478, #29630 and #34704 and Vienna Drosophila Research Center #107740), *hb*-Gal4 (Vienna Tile library [104] VT038544 and VT038545), UAS-*hb* (Bloomington Stock Center #8503), *repo*-Gal4/TM6B (kindly provided by Marion Sillies), *moody*-Gal4 ([107] kindly provided by Christian Klämbt), *DMef2*-Gal4 (Bloomington Stock Center #25756) UAS-stinger-GFP (nGFP) ([170] kindly provided by Gerd Vorbrüggen), UAS-H2B:RFP (kindly provided by Andreas Wodarz) and 20xUAS-mCD8::GFP (Bloomington Stock Center #32194). Lines expressing Gal4 under control of regulatory regions of the Hb putative target genes were obtained from Bloomington Stock Center (*ago*-Gal4 (#103–788), *brk*-Gal4 (#53707), *CadN*-Gal4 (#49660), *Dl*-Gal4 (#45495), *Fas2*-Gal4 (#48449), *kni*-Gal4 (#50246), *rho*-Gal4 (#49379), *robo3*-Gal4 (#41256), *Sox21b*-Gal4 (#39803), *Src64B*-Gal4 (#49780), *sty*-Gal4 (#104304) and *tkv*-Gal4 (#112552)).

All crosses were performed with an approximate ratio of 4:3 female:male flies. Crosses were always provided with additional yeast and were kept at 12h:12h dark:light cycle and controlled humidity, except the RNAi experiments, that were kept at 28°C and constant darkness.

#### *hb* RNA interference

We obtained 4 different UAS-*hb*^dsRNA^ lines from Bloomington Stock Center (#54478, #29630 and #34704) and from the Vienna Drosophila Research Center (#107740). To evaluate the knock-down efficiency of these RNAi lines, we took advantage of the fact that Hb is known to be necessary during early embryogenesis (Lehmann and Nüsslein-Volhard, 1987; Nüsslein-Volhard and Wieschaus, 1980). UAS-*hb*^dsRNA^ flies were crossed to the *hb*-Gal4 lines (VT038544 and VT038545) and embryonic lethality was evaluated for all four RNAi lines. Only one of the RNAi lines, namely #34704, produced no adult flies and few dead pupae when crossed with the *hb*-Gal4 flies. The other three lines produced a normal number of offspring with no obvious embryonic or larval lethality phenotype. Consequently, we used the #34704 line for the knock-down experiments in eye-antennal imaginal discs. Please note that the evaluation of knock-down efficiency in the developing eye-antennal discs using quantitative PCR is very limited because hb is only expressed in two cells in the entire disc and thus the expression is very low (practically no reads are detected by RNA-seq, see raw read counts available via GEO submission GSE94915). However, since we obtain the same qualitative phenotype on carpet cells with the RNAi experiments and the temperature sensitive mutant (see below), we are confident that the RNAi affects *hb* expression specifically.

#### *hb*^*TS*^ cross

*hb*^*TS1*^, *rsd*^*1*^*/TM3*, *Sb*^*1*^ flies (Bloomington Stock Center #1753) were crossed to *hb*^*12*^, *st*^*1*^, *e*^*1*^*/TM3*, *Sb*^*1*^ flies (Bloomington Stock Center #1755) to generate a *hb*^*TS1*^*/hb*^*12*^ stock. This line was kept at 18°C and constant light and larvae were only transferred to the restrictive temperature (28°C) for the loss of function experiments.

#### *in-situ* hybridization

Standard procedures were followed to clone a fragment (872 bp) of *hunchback* gene sequence into pCRII vector and to synthesize an antisense digoxigenin-labeled RNA probe (and sense probe for the negative control). RNA probes were hydrolyzed with Na-Carboante buffer for 30.5 minutes. Eye-antennal discs were dissected in cold PBS and fixed with 4% paraformaldehyde. Hybridization was carried out at 63°C overnight with 5 μl of RNA probe (291 ng/μl) in 50 μl of hybridization buffer. Anti-Dig antibody (1:2000, Sigma-Aldrich) was used to detect the probe and revealed with NBT/BCIP reaction mix. Pictures were taken with a Zeiss Axioplan microscope.

#### Immunohistochemistry

Antibody stainings were performed using standard procedures [171]. In all cases, dissected eye-antennal discs were fixed with 4% paraformaldehyde before incubating with primary and secondary antibodies. If not stated otherwise in the text, all eye-antennal discs were dissected from wandering late L3 larvae.

Antibodies used were: rabbit α-Repo ([143], 1:1000), guinea-pig α-Hb ([172], 1:50), mouse α-cut (Invitrogen, 1:100), rabbit α-Hb (kind gift from Chris Q. Doe, 1:100), rat α-CadN (kind gift from Dr. Marion Silies, 1:50), mouse α-Fas2 (Developmental Studies Hybridoma Bank (DSHB), 1:10), Cy3-α-HRP (kind gift from Martin Göpfert, 1:300), goat α-rabbit Alexa Fluor 488 (Invitrogen, 1:1000), goat α-rabbit Alexa Fluor 555 (Invitrogen, 1:100) and goat α-guinea-pig Alexa Fluor 555 (Invitrogen, 1:1000). A solution of 80% glycerol + 4% n-propyl gallate in PBS was used as mounting medium for all stained discs. Pictures were taken on a Zeiss LSM-510 confocal laser scanning microscope.

The area of the nuclei of different glia cells was measured from discs stained with rabbit α-Repo using ImageJ 1.48v. In each disc, the largest one or two nuclei were identified as carpet cells and measured (“one CC” and “two CCs” in Fig 5D). Additionally, four more Repo-positive glia cell nuclei in each disc were measured that were in the same region and identified as “other glia” in Fig 5D.

#### Blood-eye barrier assay

The integrity of the blood-eye barrier of *hunchback* knock-down flies was studied following the protocol from [173]. *moody*-Gal4 virgin females were crossed with UAS-*hb*^dsRNA^ males at 28°C. UAS-*hb*^dsRNA^ flies were used as control and also raised at 28°C. 2–3 day old adults from these crosses were injected in the abdomen with 3–5 kDa FITC dextran (Sigma-Aldrich) (0.3 μl the females and 0.2 μl the males of 25 mg/ml solution). Animals were allowed to recover in fresh food over-night. Only surviving animals were scored. Dye penetrance in each eye was assessed qualitatively using a LEICA M205 FA fluorescent stereo microscope.

## Supporting information

S1 Fig Multi-dimension scaling plot of RNA-seq samples. (A) Count data of all three time points (72h AEL, 96h AEL and 120h AEL). (B) Count data of only 96h AEL and 120h AEL.
(TIF)

S2 Fig Mef2 driver line expression. *Mef2*-expressing cells are visualized with a *Mef2*-Gal4 driver line crossed with UAS-H2B::RFP reporter (red).
(TIF)

S3 Fig *hb* expression in the eye-antennal disc. Different detection methods showing *hb* expression in eye-antennal discs. (A) in-situ hybridization of *hb* mRNA. *hb* is expressed in two large domains (black arrows) at the posterior end of the eye field. (B) Antibody staining of Hb protein (rabbit α-Hb) in late L3 eye-antennal discs. Co-staining with Phalloidin (B’, B”) shows that the Hb positive cells are located between the photoreceptor axons on their way to the optic stalk. (C) Antibody staining of Hb protein (rabbit α-Hb) in late L2 eye-antennal imaginal discs. (D) Expression of histone-bound RFP (UAS-H2B::RFP) driven by VT038544 line (*hb*-Gal4) containing an enhancer region near the *hb* locus. (E) Expression of histone-bound RFP (UAS-H2B::RFP) driven by VT038545 line (*hb*-Gal4) containing an enhancer region near the *hb* locus. (VT038544-Gal4 and VT038545-Gal4 driver lines were obtained from the Vienna Tile collection, see S4 Fig for details). In all pictures, anterior is to the right. Eye disc (ed), optic stalk (os). Scale bar = 20 μm.
(TIF)

S4 Fig Genomic location of Vienna Tile *hb* driver lines. Arrows indicate the regions used to drive *hb* expression with Gal4 system. Bellow, are colored tracks provided by the BDTNP project [83] showing open chromatin profiles and transcription factor binding. The last black tracks show sequence conservation across different insect species. These tracks were visualized using UCSC Browser [168].
(TIF)

S5 Fig The strength of the effect of loss of Hb function in carpet cells is not significantly different at different time points. (A) A significant difference in the distribution of the number of polyploid glia cells in *hb*^*TS*^ flies is only observed between raising larvae at the restrictive temperature 48h AEL and 72h AEL. However, this difference is also significant in the wild type (WT). This can be due to the fact that more larvae die when transferred to the restrictive temperature too early (at 24h AEL or 48h AEL). (B) Pearson’s Chi-squared test was performed to determine if the distribution of the different number of cells (0, 1 or 2) was equal across the time points for the same conditions (WT or *hb*^*TS*^). *: p-val < 0.05, ***: p-val < 0.0005.
(TIF)

S6 Fig Expression of Hb targets CadN (rat α-CadN), Fas2 (mouse α-Fas2) and *brk* (*brk*-Gal4 #53707) in late L3 eye-antennal imaginal discs. (TIF)

S1 Table Significantly enriched GO terms in the expression clusters. (XLSX)

S2 Table Significantly enriched transcription factors in the expression clusters. (XLSX)

S3 Table Cluster assignment of So and Eya target genes. (XLSX)

S4 Table Putative Hb target genes differentially expressed. Table contains two sheets: first sheet lists putative Hb targets differentially expressed between 72h AEL and 96h AEL and second sheet lists the differentially expressed genes between 96h AEL and 120h AEL. “Instances”: number of Hb motifs found ±1000 bp from TTS. Right-side table shows how many of these genes belong to each cluster and the percentage over the total number of genes in that cluster.
(XLSX)

S5 Table Putative Hb target genes in clusters 12 and 13. Table contains three sheets: first sheet contains the gene ID, name and symbol of the 77 genes, and the cluster they belong to; second sheet lists the GO terms associated to each of the 77 genes; third sheet contains the number of times each GO term appears in the second sheet.
(XLSX)

S6 Table Results of Hb motif search in sequences covered by the used Gal4 driver lines. (DOCX)
