# Supplementary figures and images for: Dynamic genome wide expression profiling of Drosophila head development reveals a novel role of Hunchback in retinal glia cell development and blood-brain barrier integrity
Source: PLoS Genet. 2018 Jan 23;14(1):e1007180. doi: 10.1371/journal.pgen.1007180 (PMC5796731; doi:10.1371/journal.pgen.1007180)

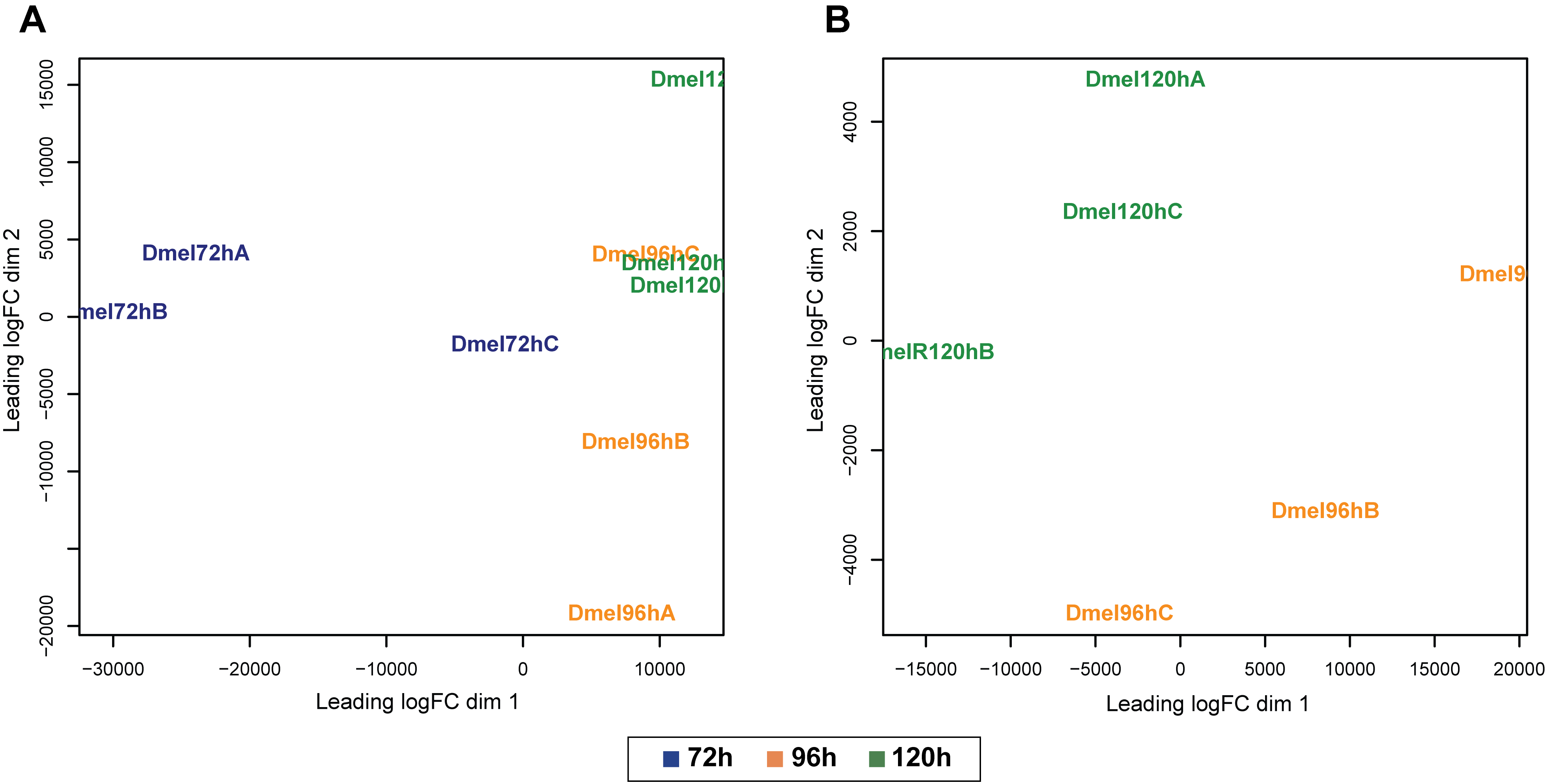

Supplement: S1 Fig — (A) Count data of all three time points (72h AEL, 96h AEL and 120h AEL). (B) Count data of only 96h AEL and 120h AEL. (TIF) [file pgen.1007180.s001.tif]

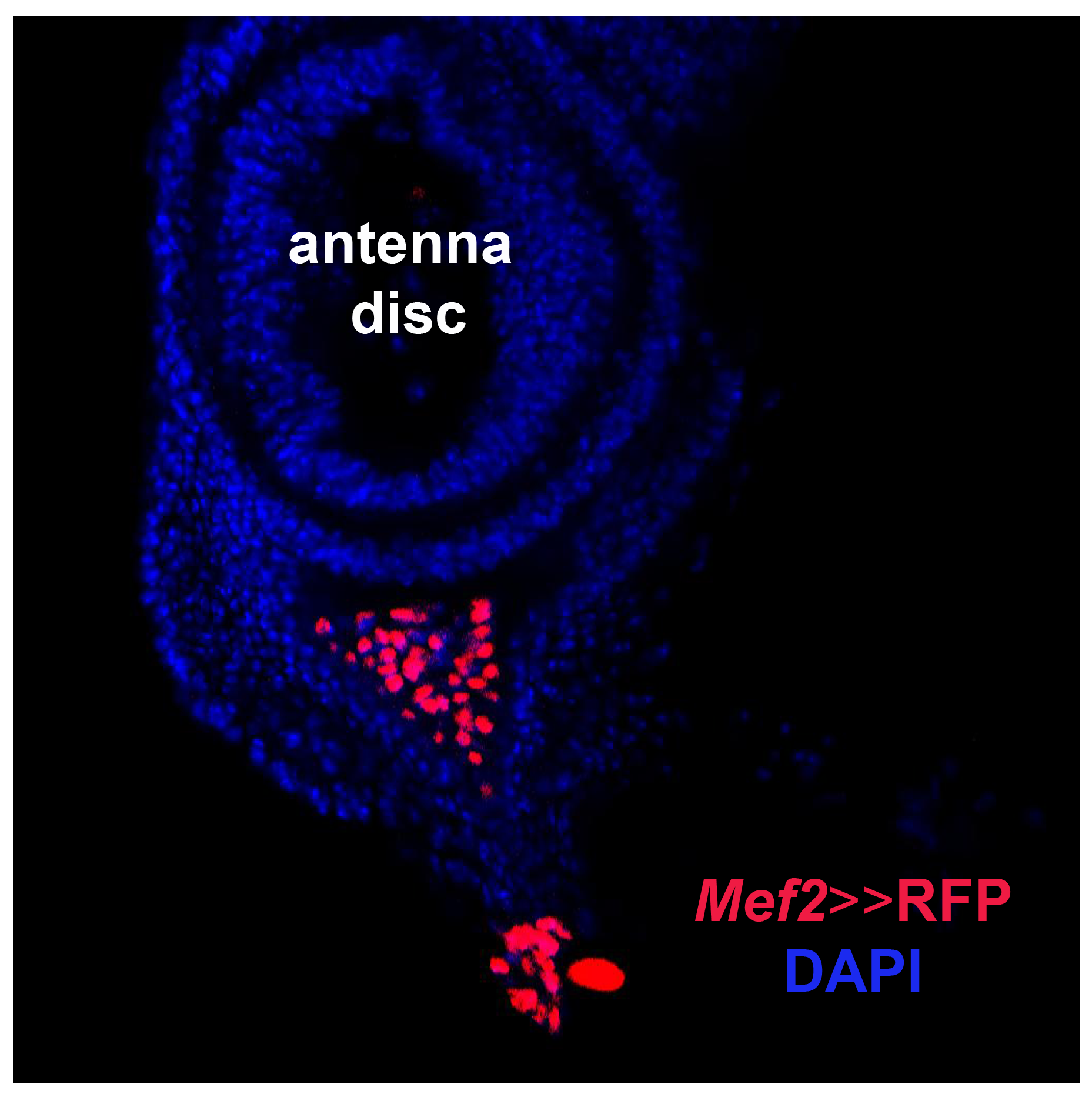

Supplement: S2 Fig — Mef2-expressing cells are visualized with a Mef2-Gal4 driver line crossed with UAS-H2B::RFP reporter (red). (TIF) [file pgen.1007180.s002.tif]

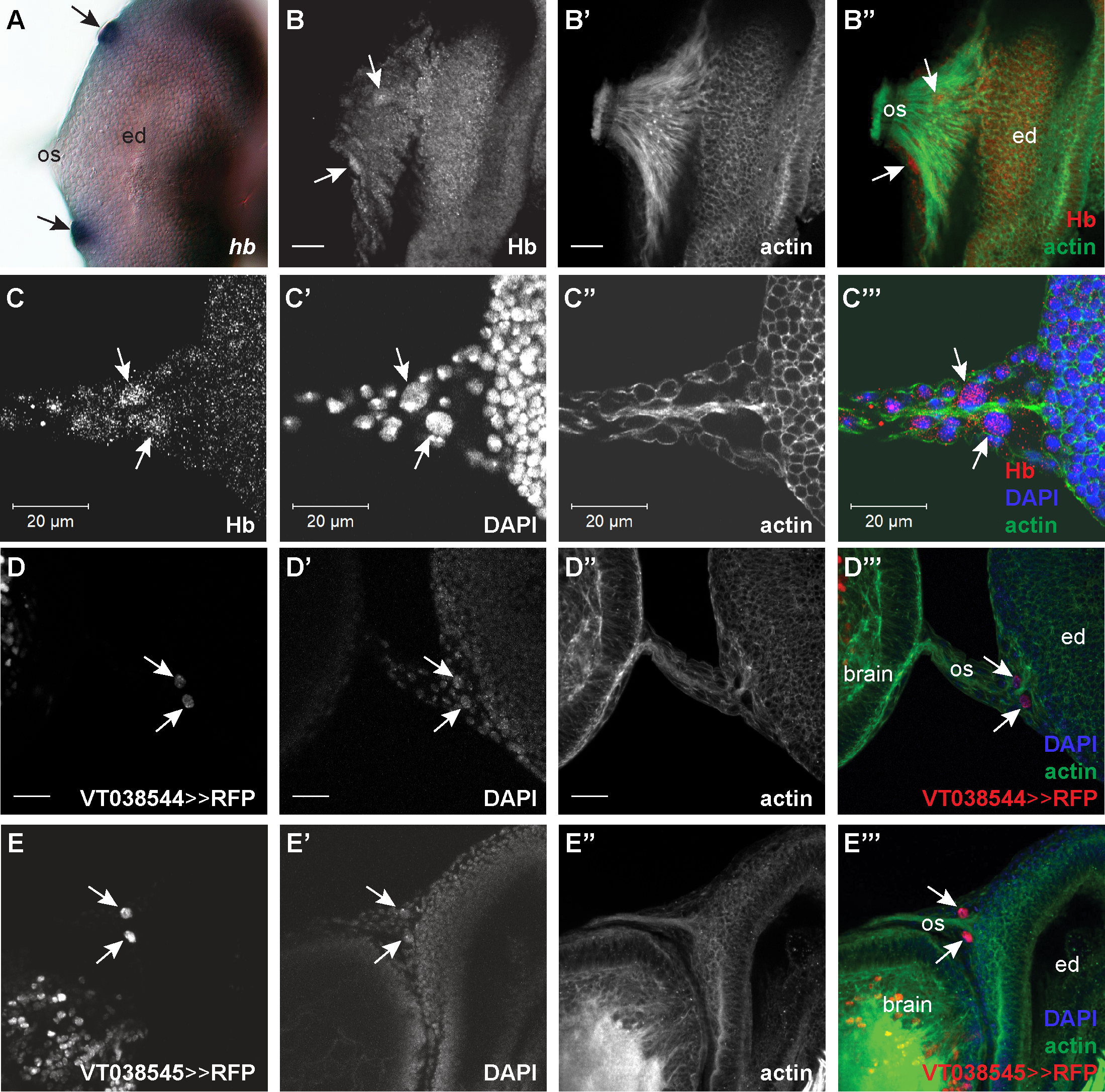

Supplement: S3 Fig — Different detection methods showing hb expression in eye-antennal discs. (A) in-situ hybridization of hb mRNA. hb is expressed in two large domains (black arrows) at the posterior end of the eye field. (B) Antibody staining of Hb protein (rabbit α-Hb) in late L3 eye-antennal discs. Co-staining with Phalloidin (B’, B”) shows that the Hb positive cells are located between the photoreceptor axons on their way to the optic stalk. (C) Antibody staining of Hb protein (rabbit α-Hb) in late L2 eye-antennal imaginal discs. (D) Expression of histone-bound RFP (UAS-H2B::RFP) driven by VT038544 line (hb-Gal4) containing an enhancer region near the hb locus. (E) Expression of histone-bound RFP (UAS-H2B::RFP) driven by VT038545 line (hb-Gal4) containing an enhancer region near the hb locus. (VT038544-Gal4 and VT038545-Gal4 driver lines were obtained from the Vienna Tile collection, see S4 Fig for details). In all pictures, anterior is to the right. Eye disc (ed), optic stalk (os). Scale bar = 20 μm. (TIF) [file pgen.1007180.s003.tif]

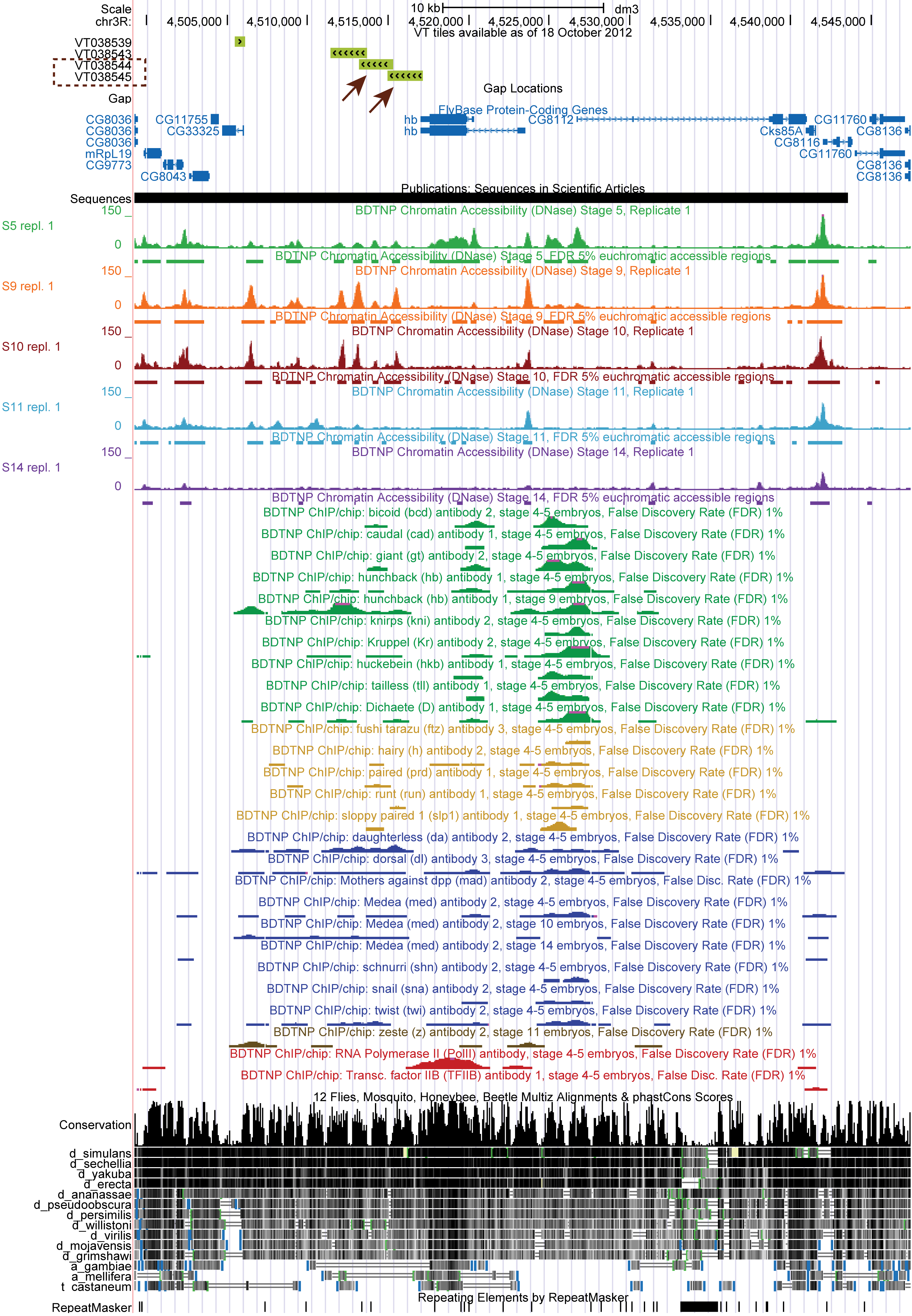

Supplement: S4 Fig — Arrows indicate the regions used to drive hb expression with Gal4 system. Bellow, are colored tracks provided by the BDTNP project [83] showing open chromatin profiles and transcription factor binding. The last black tracks show sequence conservation across different insect species. These tracks were visualized using UCSC Browser [168]. (TIF) [file pgen.1007180.s004.tif]

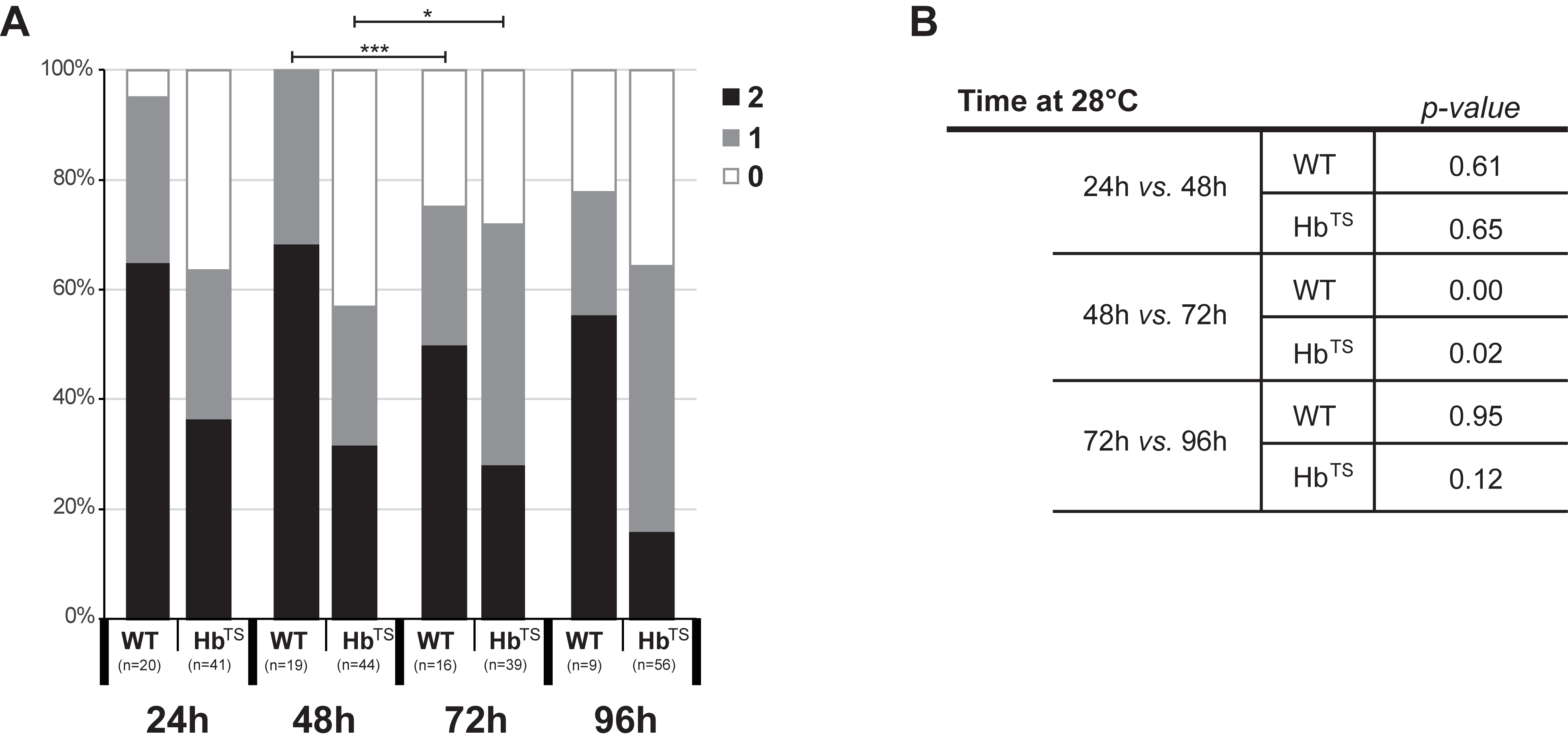

Supplement: S5 Fig — (A) A significant difference in the distribution of the number of polyploid glia cells in hbTS flies is only observed between raising larvae at the restrictive temperature 48h AEL and 72h AEL. However, this difference is also significant in the wild type (WT). This can be due to the fact that more larvae die when transferred to the restrictive temperature too early (at 24h AEL or 48h AEL). (B) Pearson’s Chi-squared test was performed to determine if the distribution of the different number of cells (0, 1 or 2) was equal across the time points for the same conditions (WT or hbTS). *: p-val < 0.05, ***: p-val < 0.0005. (TIF) [file pgen.1007180.s005.tif]

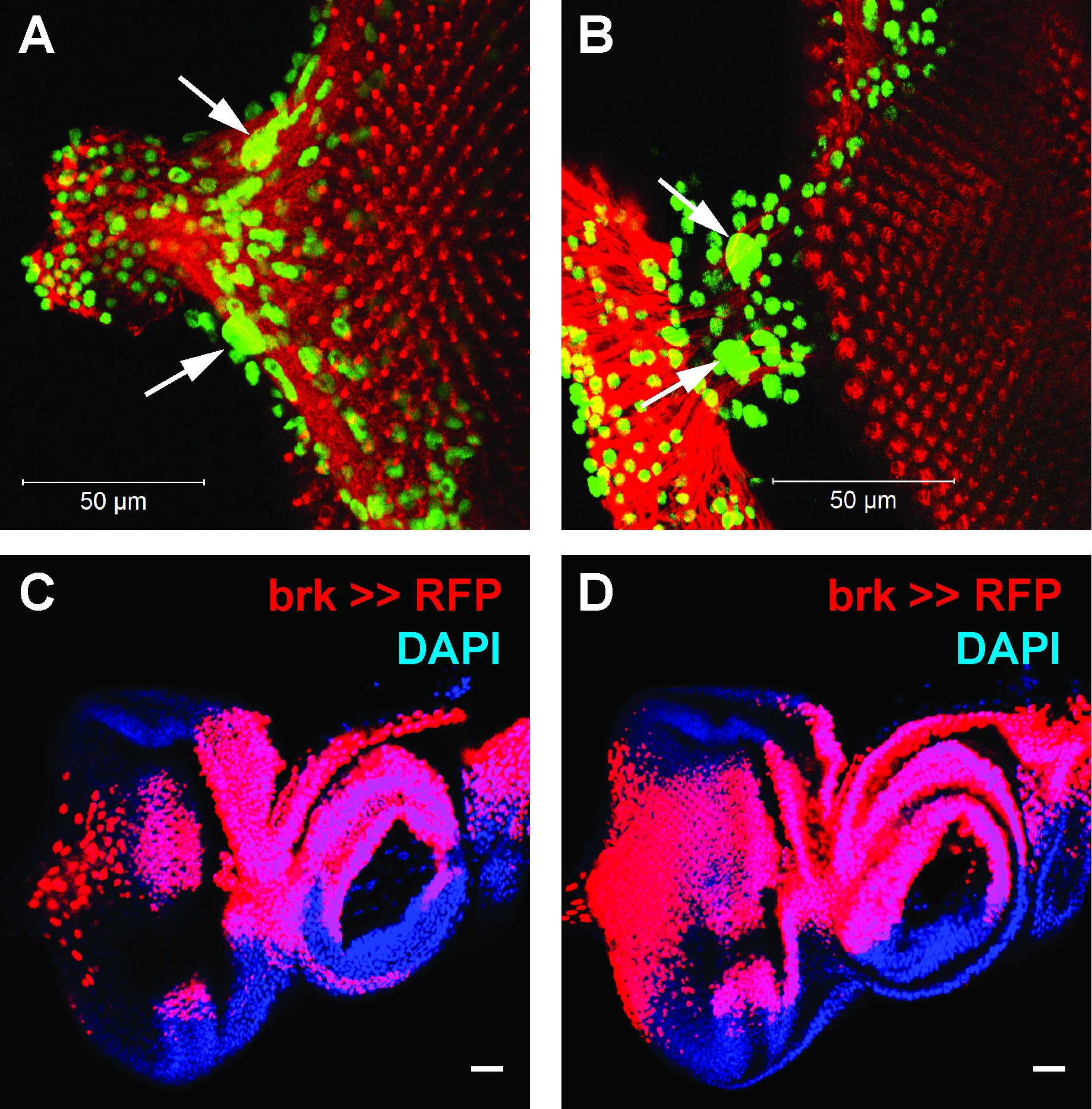

Supplement: S6 Fig — (TIF) [file pgen.1007180.s006.tif]
