# Supplementary material for: Dynamic genome wide expression profiling of Drosophila head development reveals a novel role of Hunchback in retinal glia cell development and blood-brain barrier integrity
Source: PLoS Genet. 2018 Jan 23;14(1):e1007180. doi: 10.1371/journal.pgen.1007180 (PMC5796731; doi:10.1371/journal.pgen.1007180)
Supplement: S6 Table — (DOCX) [file pgen.1007180.s012.docx]

**Fas2:**

- #48449 (GMR11C08; X:4061472..4064988) has a Hb target motif with:

| 1 putative sites were predicted with these settings (99%) in sequence named **dm6_ct_coords_7288** | | | | | | | |
| --- | --- | --- | --- | --- | --- | --- | --- |
| Model ID | Model name | Score | Relative score | Start | End | Strand | predicted site sequence |
| MA0049.1 | hb | 12.824 | 1.00001474388335 | 3417 | 3426 | -1 | GCATAAAAAA |

**CadN:**

- #49660 (GMR31A11; 2L:17665454..17666638) has two Hb target motifs with:

| 2 putative sites were predicted with these settings (93%) in sequence named **dm6_dna** | | | | | | | |
| --- | --- | --- | --- | --- | --- | --- | --- |
| Model ID | Model name | Score | Relative score | Start | End | Strand | predicted site sequence |
| MA0049.1 | hb | 10.601 | 0.931525897820554 | 406 | 415 | 1 | GCATTAAAAA |
| MA0049.1 | hb | 10.724 | 0.935315429114177 | 1088 | 1097 | 1 | GAATAAAAAC |
